# Alternative processing of human *HTT* mRNA with implications for Huntington’s disease therapeutics

**DOI:** 10.1093/brain/awac241

**Published:** 2022-07-06

**Authors:** Sandra Fienko, Christian Landles, Kirupa Sathasivam, Sean J McAteer, Rebecca E Milton, Georgina F Osborne, Edward J Smith, Samuel T Jones, Marie K Bondulich, Emily C E Danby, Jemima Phillips, Bridget A Taxy, Holly B Kordasiewicz, Gillian P Bates

**Affiliations:** Department of Neurodegenerative Disease, Huntington’s Disease Centre and UK Dementia Research Institute at UCL, Queen Square Institute of Neurology, UCL, London WC1N 3BG, UK; Department of Neurodegenerative Disease, Huntington’s Disease Centre and UK Dementia Research Institute at UCL, Queen Square Institute of Neurology, UCL, London WC1N 3BG, UK; Department of Neurodegenerative Disease, Huntington’s Disease Centre and UK Dementia Research Institute at UCL, Queen Square Institute of Neurology, UCL, London WC1N 3BG, UK; Department of Neurodegenerative Disease, Huntington’s Disease Centre and UK Dementia Research Institute at UCL, Queen Square Institute of Neurology, UCL, London WC1N 3BG, UK; Department of Neurodegenerative Disease, Huntington’s Disease Centre and UK Dementia Research Institute at UCL, Queen Square Institute of Neurology, UCL, London WC1N 3BG, UK; Department of Neurodegenerative Disease, Huntington’s Disease Centre and UK Dementia Research Institute at UCL, Queen Square Institute of Neurology, UCL, London WC1N 3BG, UK; Department of Neurodegenerative Disease, Huntington’s Disease Centre and UK Dementia Research Institute at UCL, Queen Square Institute of Neurology, UCL, London WC1N 3BG, UK; Department of Neurodegenerative Disease, Huntington’s Disease Centre and UK Dementia Research Institute at UCL, Queen Square Institute of Neurology, UCL, London WC1N 3BG, UK; Department of Neurodegenerative Disease, Huntington’s Disease Centre and UK Dementia Research Institute at UCL, Queen Square Institute of Neurology, UCL, London WC1N 3BG, UK; Department of Neurodegenerative Disease, Huntington’s Disease Centre and UK Dementia Research Institute at UCL, Queen Square Institute of Neurology, UCL, London WC1N 3BG, UK; Department of Neurodegenerative Disease, Huntington’s Disease Centre and UK Dementia Research Institute at UCL, Queen Square Institute of Neurology, UCL, London WC1N 3BG, UK; Department of Neurodegenerative Disease, Huntington’s Disease Centre and UK Dementia Research Institute at UCL, Queen Square Institute of Neurology, UCL, London WC1N 3BG, UK; Ionis Pharmaceuticals, Carlsbad, CA 92008, USA; Department of Neurodegenerative Disease, Huntington’s Disease Centre and UK Dementia Research Institute at UCL, Queen Square Institute of Neurology, UCL, London WC1N 3BG, UK

**Keywords:** Huntington’s disease, YAC128 mice, nuclear RNA clusters, *HTT1a* transcript, exon 1 HTT and aggregation

## Abstract

Huntington disease is caused by a CAG repeat expansion in exon 1 of the huntingtin gene (*HTT*) that is translated into a polyglutamine stretch in the huntingtin protein (HTT). We previously showed that *HTT* mRNA carrying an expanded CAG repeat was incompletely spliced to generate *HTT1a*, an exon 1 only transcript, which was translated to produce the highly aggregation-prone and pathogenic exon 1 HTT protein. This occurred in all knock-in mouse models of Huntington’s disease and could be detected in patient cell lines and post-mortem brains. To extend these findings to a model system expressing human *HTT*, we took advantage of YAC128 mice that are transgenic for a yeast artificial chromosome carrying human *HTT* with an expanded CAG repeat.

We discovered that the *HTT1a* transcript could be detected throughout the brains of YAC128 mice. We implemented RNAscope to visualize *HTT* transcripts at the single molecule level and found that full-length *HTT* and *HTT1a* were retained together in large nuclear RNA clusters, as well as being present as single transcripts in the cytoplasm. Homogeneous time-resolved fluorescence analysis demonstrated that the *HTT1a* transcript had been translated to produce the exon 1 HTT protein. The levels of exon 1 HTT in YAC128 mice, correlated with HTT aggregation, supportive of the hypothesis that exon 1 HTT initiates the aggregation process.

Huntingtin-lowering strategies are a major focus of therapeutic development for Huntington’s disease. These approaches often target full-length *HTT* alone and would not be expected to reduce pathogenic exon 1 HTT levels. We have established YAC128 mouse embryonic fibroblast lines and shown that, together with our QuantiGene multiplex assay, these provide an effective screening tool for agents that target *HTT* transcripts. The effects of current targeting strategies on nuclear RNA clusters are unknown, structures that may have a pathogenic role or alternatively could be protective by retaining *HTT1a* in the nucleus and preventing it from being translated. In light of recently halted antisense oligonucleotide trials, it is vital that agents targeting *HTT1a* are developed, and that the effects of *HTT*-lowering strategies on the subcellular levels of all *HTT* transcripts and their various HTT protein isoforms are understood.

## Teaser

Fienko *et al.* show that in YAC128 mice, human huntingtin (*HTT*) mRNA undergoes alternative processing to generate *HTT1a*, which is retained in the nucleus within RNA clusters or translated to generate the highly pathogenic exon 1 HTT protein. These findings have implications for the design of huntingtin-lowering therapies.

## Introduction

Huntington’s disease is an hereditary neurodegenerative disorder that manifests with movement disturbances, psychiatric changes and cognitive decline.^1^ It is caused by an unstable CAG repeat expansion in exon 1 of the huntingtin gene (*HTT*), that is translated to produce an unusually long polyglutamine (polyQ) stretch in the huntingtin protein (HTT).^2^ Mutant HTT self-associates to form aggregates that are deposited as inclusion bodies in patient brains,^3^ and the neuropathology of Huntington’s disease is also marked by synaptic death and neuronal cell loss in the striatum, cortex and other brain regions.^4,5^ The treatments that are currently available for Huntington’s disease focus on managing symptoms, because a disease-modifying therapy to delay the onset or slow the progression of the disease does not exist.

The *HTT* gene contains 67 exons and encodes a 350-kDa protein.^2^ Thus far, three full-length *HTT* mRNA isoforms have been described that are produced by alternative polyadenylation in the 3′ untranslated region (3′ UTR).^6^ We have previously shown that, in the context of an expanded CAG repeat, two small transcripts that contain exon 1 and 5′ intron 1 sequences (*HTT1a*) are produced by the incomplete splicing of the *HTT* transcript.^7,8^ This occurs when one of two cryptic polyadenylation (polyA) signals in intron 1 becomes activated. These cryptic polyA signals extend 2710 and 7327 bp into intron 1 for human *HTT*^7,8^ and 680 and 1145 bp into intron 1 for mouse *Htt*.^7^ The extent to which *HTT* is subjected to this alternative mRNA processing event is dictated by CAG repeat length, in that the longer the repeat the more *HTT1a* is generated.^7^ Importantly, the *HTT1a* mRNA is translated to produce the highly pathogenic and aggregation-prone exon 1 HTT protein.^9,10^

In recent years various therapeutic strategies have been devised to combat Huntington’s disease. While therapies aimed at restoring defective molecular pathways or clearing protein aggregates have received consideration,^11–14^ strategies to lower *HTT* mRNA are now a focal point for Huntington’s disease therapeutics. Methods that reduce *HTT* mRNA levels and/or lead to translational suppression, thereby halting pathogenic protein production, include antisense oligonucleotides (ASOs), interfering RNAs (RNAi), zinc finger proteins (ZFPs) and small molecule mRNA splicing modulators.^15^ ASO-based therapies aiming to reduce either full-length wild-type and mutant *HTT* mRNAs (tominersen, Roche) or specifically mutant *HTT* mRNA (Wave Life Sciences)^16^ had progressed to phase III and phase I/II clinical trials, respectively. However, in 2021 both trials were halted as the drugs failed to either show higher efficacy over placebo, as was the case for tominersen, or to significantly lower *HTT* mRNA levels, as reported for the allele-selective ASOs.^16^ Although multiple factors could have contributed to the failure of these ASOs, it is important to highlight that both therapies targeted only full-length *HTT* transcripts, leaving the *HTT1a* mRNA intact.

Mouse models that express a mutant version of the entire human *HTT* gene in the form of a yeast artificial chromosome (YAC)^17^ or bacterial artificial chromosome (BAC)^18^ can be used to validate therapeutic approaches targeting human *HTT* transcripts.^19^ YAC128 mice are transgenic for full-length human *HTT* with an expanded CAG repeat encoding 125 glutamines.^17^ To extend our understanding of *HTT* expression in this mouse line, we have performed a detailed analysis of huntingtin at both the RNA and protein levels. Our findings indicate that the human *HTT* mRNA in YAC128 mice undergoes alternative processing throughout the brain to produce *HTT1a*. This small transcript was either retained in nuclear RNA clusters, where it co-localized with full-length *HTT*, or was exported to the cytoplasm and translated to produce the exon 1 HTT protein. In young mice, aggregated HTT levels were greatest in the cerebellum, the region containing the highest level of exon 1 HTT, consistent with the aggregation-prone nature of this mutant form of HTT. Both the formation of RNA clusters and the generation of the exon 1 HTT protein have unexplored implications for development of therapies for Huntington’s disease.

## Materials and methods

### Ethics statement

All procedures were performed in accordance with the Animals (Scientific Procedures) Act, 1996, and approved by the University College London Ethical Review Process Committee.

### Animal colony breeding and maintenance

YAC128 and zQ175 mice were maintained on a C57BL6/J (Charles River) background. Mice were group-housed depending on gender, and genotypes were mixed within cages. All animals were kept in individually ventilated cages containing Aspen Chips 4 Premium bedding (Datesand) with environmental enrichment in the form of chew sticks and a play tunnel (Datesand). All mice had *ad libitum* access to water and chow (Teklad global 18% protein diet, Envigo, the Netherlands). The temperature was automatically regulated at 21°C ± 1°C and animals were kept on a 12-h light/dark cycle. The animal facility was barrier-maintained and quarterly non-sacrificial FELASA (Federation of European Laboratory Animal Science Associations) screens found no evidence of pathogens. Animals were sacrificed at 2, 3, 5, 6, 9 and 12 months of age, brains rapidly dissected, frozen in liquid nitrogen and stored at −80°C.

### DNA extractions, genotyping and repeat sizing

Genomic DNA was extracted from ear notches^20^ and genotyping was performed as previously described.^21^ The polyQ repeat of 125 glutamines in YAC128 mice is encoded by (CAG)_23_(CAA)_3_CAGCAA(CAG)_80_(CAA)_3_CAGCAA(CAG)_10_CAACAG, which is stable on germline transmission.^22^ The zQ175 mice had CAG repeat expansions of 205.

### Cell culture and antisense oligonucleotide transfection

The procedures used for the isolation of mouse embryonic fibroblasts (MEFs), their transformation with the Simian Virus-40 large tumour antigen (SV40 T-antigen) and their transfection with ASOs is outlined in detail in the Supplementary material.

### RNA extraction and cDNA synthesis

Brain tissue was homogenized in Qiazol lysis reagent for 20–30 s. The homogenizing probe was washed with 100% ethanol and ddH_2_O between each sample. Total RNA was extracted from brain tissue and MEFs using the RNeasy mini-kit (Qiagen) following the manufacturer’s recommended protocol. DNase I treatment was carried out using the RNA-free DNase kit (Qiagen), followed by RNA elution in the suitable amount of nuclease-free H_2_O (Sigma-Aldrich). The quality and quantity of RNA was determined by NanoDrop 1000 (Thermo Fisher Scientific). cDNA synthesis was performed with 1 μg RNA using M-MLV reverse transcriptase (Invitrogen) and oligo-dT_(18)_ primers (Invitrogen) according to the manufacture’s recommendation. A negative control was always included, to which no reverse transcriptase was added (−RT).

### Relative real-time quantitative PCR

Primers and probes (Taqman assays) used for the quantification of *HTT* transcripts were purchased from Eurofins, whereas mouse reference gene assays were from Thermo Fisher Scientific. The amplification efficiencies of primer sets were assessed by 4-fold serial dilutions of a pool of equal volumes of cDNA from six YAC128 animals and are listed in Supplementary Table 1. The amplification efficiencies of the real-time quantitative PCR (qPCR) assays were assessed by 4-fold serial dilution of cDNA from the cortex of 2-month-old YAC128 animals. After determination of the linear range of the qPCR reaction, a 1:10 dilution of the cDNA was chosen for all brain regions as well as MEFs. Each 15 μl reaction consisted of 3 μl diluted cDNA, TaqMan Fast Advanced Mix (Applied Biosystems) as well as the appropriate gene expression assay, distributed into 96-well thin wall Hard-Shell PCR plates (Bio-Rad). Plates were sealed with Microseal ‘B’ seals (Bio-Rad), centrifuged at 800*g* for 30 s and run in a Bio-Rad CFX96 qPCR machine as follows: 40 s at 95°C, 40× [7 s at 95°C, 20 s at 60°C (or 65°C for 3′ UTR)] (Supplementary Table 1). Three technical replicates of each biological sample were used for all assays. C*q* values deviating by 0.5 from the mean were excluded from further analysis. Data for genes of interest were normalized to the geometric mean of the reference genes (*Sdha*, *Ubc* and *Atp5b*) and the 2^−ΔCt2^ was calculated.^23^

### Tissue lysis and QuantiGene assays

Brain samples and cell lysates were prepared as described previously.^20,24^ To assess the linearity of the QuantiGene probe set, equal volumes of brain homogenates or cell lysates of the same genotype were pooled and a 2-fold serial dilution was prepared for each pool. Subsequently, the brain homogenates or cell lysates were diluted so that the fluorescent signal for all probe sets in the QuantiGene panel fell within the linear range. The QuantiGene plex was assayed in duplicate (brain tissue) or quadruplicate (cultured cells) as per the manufacturer’s protocol with the exception that the streptavidin *R*–phycoerythrin conjugate (SAPE) was incubated at 51°C. Plates were read on a Magpix (Luminex). After background subtraction, the median fluorescence intensity for the genes of interest was normalized to the geometric mean of the median fluorescent intensity for the reference genes (Supplementary Table 2).

### RNAscope analysis and quantification

Procedures for RNAscope probe hybridizations are outlined in detail in the Supplementary material.

### Antibodies

All antibodies are listed in Supplementary Table 3.

### Immunohistochemistry

Immunohistochemistry was performed as described in detail elsewhere.^25^ The S830 primary antibody was applied at a 1:3000 dilution. Images were acquired with a Zeiss AxioSkop2 plus microscope fitted with a Zeiss AxioCam HRc colour camera using Zeiss AxioVision 4.7 software.

### Immunoprecipitation and western blotting

Immunoprecipitation from wild-type and YAC128 aged cortical samples was performed using the anti-polyglutamine 3B5H10 antibody (Sigma-Aldrich) exactly as described.^7,26^ For western blotting, immunoprecipitations were denatured, separated by 10% sodium dodecyl sulphate–polyacrylamide gel electrophoresis (SDS-PAGE), blotted onto nitrocellulose membrane and detected by chemiluminescent detection, exactly as described.^25,26^ Antibody dilutions were, anti-HTT S830 (sheep): 1:2000 and anti-HTT (rabbit) CHDI-90000148: 1:1000.

### Homogeneous time-resolved FRET

Homogeneous time-resolved FRET (HTRF) was performed as previously described.^21^ A detailed account of the optimization and use of assays for YAC128 tissues and MEFs is provided in the Supplementary material.

### Behavioural and phenotype assessment

Weight gain, grip strength, rotarod performance and locomotor activity were measured in YAC128 males (*n* = 11) and females (*n* = 10) and their wild-type littermate males (*n* = 9) and females (*n* = 10) monthly from 2 to 12 months of age. Body weight was recorded weekly to the nearest 0.1 g. Rotarod performance and grip strength measures were performed as described elsewhere.^27^ Exploratory activity in the open field was measured as described previously^28^ and detailed below. Mice were individually placed in a white open field arena (50 cm × 50 cm × 50 cm, Engineering & Design Plastics Ltd) for 30 min. Behaviour was videotaped via a camera placed above the apparatus. Activity (distance travelled, cm) was tracked and analysed using EthoVision 11.5 XT software (Noldus, The Netherlands). The arena was cleaned using 70% industrial methylated sprits between trials. The order in which mice were assessed for behavioural measures was mixed for genotype and gender and operators were blind to genotype.

### Statistical analysis

Data were screened for outliers using the ROUT test (Q = 10%; GraphPad Prism v8) and outliers were removed from the analysis. Statistical analysis was by one- or two-way ANOVA with either Tukey’s, Dunnet’s or Bonferroni *post hoc* tests. For behavioural analysis all data were screened for statistical outliers using ROUT test (Q = 10%; GraphPad Software v8, California, USA) and outliers were removed before between-group comparisons. Statistical analysis was performed with SPSS (v26; IMB, Portsmouth, UK) using GLM ANOVA or ANCOVA (body weight as a covariant), with Bonferroni *post hoc* tests. Graphs were prepared using Prism v8 (GraphPad Software, California, USA). *P*-values < 0.05 were considered statistically significant.

### Data availability

The authors confirm that all the data supporting the findings of this study are available within the article and its Supplementary material. Raw data will be shared by the corresponding author on request.

## Results

### *HTT1a* is generated in the brains of YAC128 mice through alternative RNA processing

**Figure 1:**
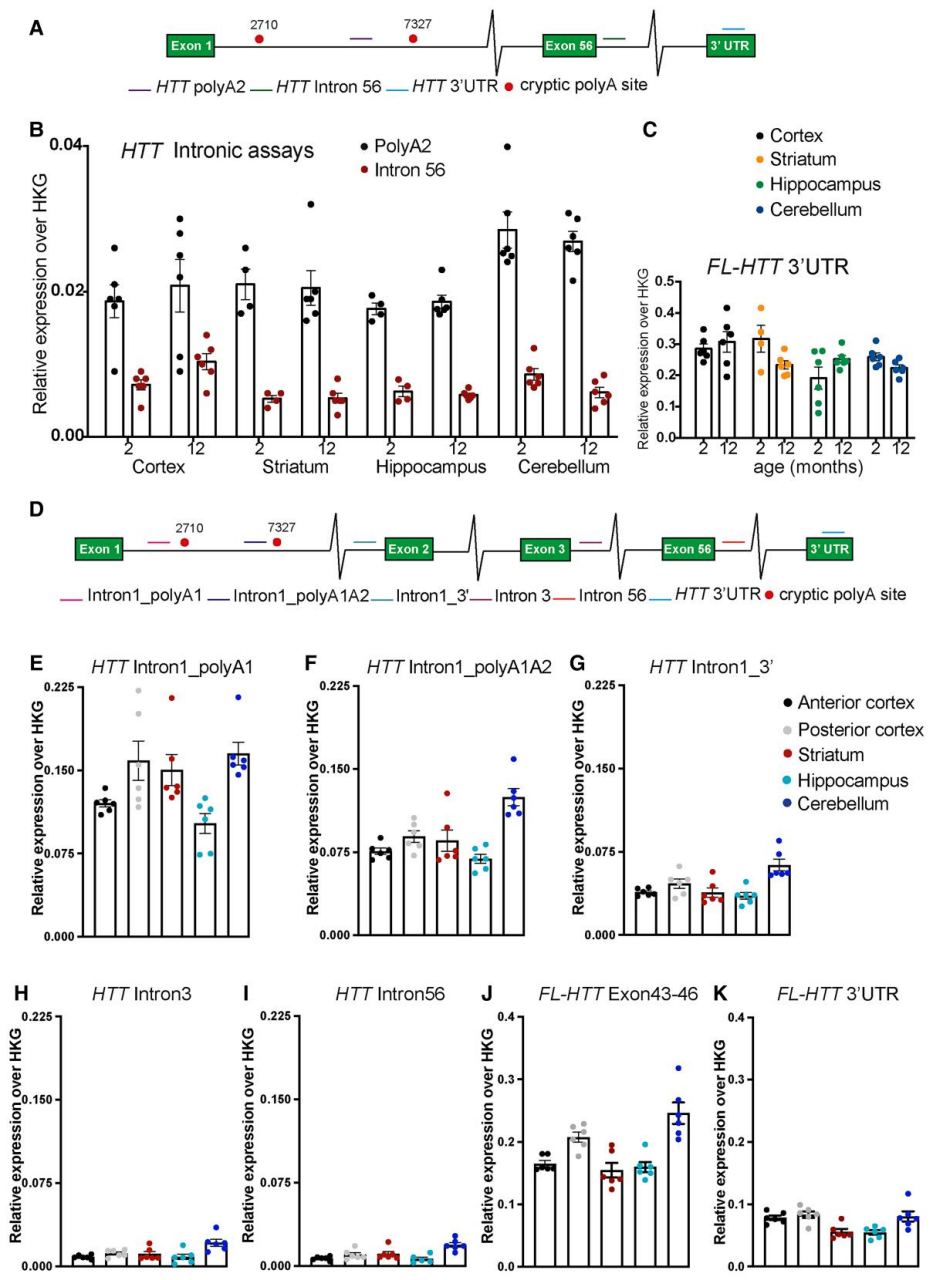
**Quantitative PCR and QuantiGene analysis of full-length *HTT* and *HTT1a* transcripts in YAC128 brains.** (**A**) Schematic of the location of the qPCR assays on the human *HTT* transcript. (**B**) *HTT1a* was detected in the cortex, striatum, hippocampus and cerebellum of 2- and 12-month-old YAC128 mice (*n* = 4–6/genotype). (**C**) Full-length *HTT* levels in the cortex, striatum, hippocampus and cerebellum as measured using the *HTT*_ 3′ UTR assay (*n* = 4–6/genotype). (**D**) Schematic of the location of the QuantiGene probe sets on the human *HTT* transcript. (**E**–**I**) Comparison of *HTT* intronic sequences between brain regions. *HTT1a* was detected by QuantiGene in the cortex, striatum, hippocampus and cerebellum of 2-month-old YAC128 mice (*n* = 4–6/genotype). (**J** and **K**) Full-length *HTT* detected by QuantiGene in the anterior and posterior cortex as well as in the striatum, hippocampus and cerebellum of 2-month-old YAC128 mice (*n* = 4–6/genotype). Two different QuantiGene probe sets targeting exons 43–46 or the 3′ UTR were designed to detect FL-*HTT*. The results are plotted on separate graphs as they render different signals due to the assay design.

We have previously shown that mouse *Htt* transcripts carrying expanded CAG repeats are alternatively processed to generate *Htt1a* in all Huntington’s disease knock-in mouse models.^7,29^ YAC128 mice express human *HTT* with an expanded CAG repeat and, using 3′ RACE (rapid amplification of cDNA ends), we showed that cryptic polyA sites at 2710 and 7327 bp within intron 1 of human *HTT* in YAC128 brains had been activated, suggesting that human *HTT1a* was generated in this mouse model.^7^ To extend these findings, we set out to track the expression profile of full-length *HTT* (*FL*-*HTT*) and *HTT1a* in the brains of YAC128 mice by qPCR. To identify the *HTT1a* transcript we applied the qPCR assay (PolyA_2_) that binds to intron 1 before the second cryptic poly(A) site at 7327 bp.^8^ We designed an assay to the 3′ UTR of *HTT* to quantify *FL-HTT* and one to intron 56 to detect any contaminating preprocessed mRNA (Fig. 1A and Supplementary Table 1). We interrogated four brain regions: cortex, striatum, hippocampus and cerebellum from YAC128 mice at 2 and 12 months of age. The *HTT1a* transcript could be readily detected in all brain regions (Fig. 1B). Quantitation of *HTT* intron 56 demonstrated that there was minimal contamination with unprocessed mRNA (Fig. 1B). The expression profile of both *HTT* transcripts, *FL-HTT* and *HTT1a* showed no consistent age-dependent changes in any of the brain regions (Fig. 1B and C).

Next, we designed a multiplex QuantiGene panel that would allow us to detect all human *HTT* transcripts simultaneously without the need for RNA extraction or error-prone cDNA synthesis. The 15-plex QuantiGene assay consisted of probes to intron 1 of human *HTT* before the first cryptic poly(A) site at 2710 bp (Intron1polyA_1_), to intron 1 before the second cryptic poly(A) site at 7327 bp (Intron1polyA_1_A _2_), to sequences at the 3′ end of intron 1 (Intron1_3′), within intron 3 (Intron 3) and within intron 56 (Intron 56), as well as to exons 43–46 and the 3′ UTR for the fully spliced coding sequence (Fig. 1D and Supplementary Table 2). The QuantiGene plex was used to determine the comparative levels of each of these *HTT* transcripts between the cortex, striatum, hippocampus and cerebellum of 2-month-old YAC128 mice. In agreement with our qPCR data, the *HTT1a* mRNA was detected in all brain regions, and the pre-mRNA intron 3 and intron 56 probe-sets gave very low background levels (Fig. 1E–I). As these assays were not quantitative, the intensities obtained for the exonic and intronic *HTT* probe-sets, within a brain region, cannot be interpreted as relative expression levels. This is exemplified by the two probe-sets that detected *FL-HTT*, which gave different median fluorescent intensity values (Fig. 1J and K), reflecting variation in probe hybridization efficiencies.

### *HTT1a* is retained in nuclear RNA clusters in YAC128 brains

**Figure 2:**
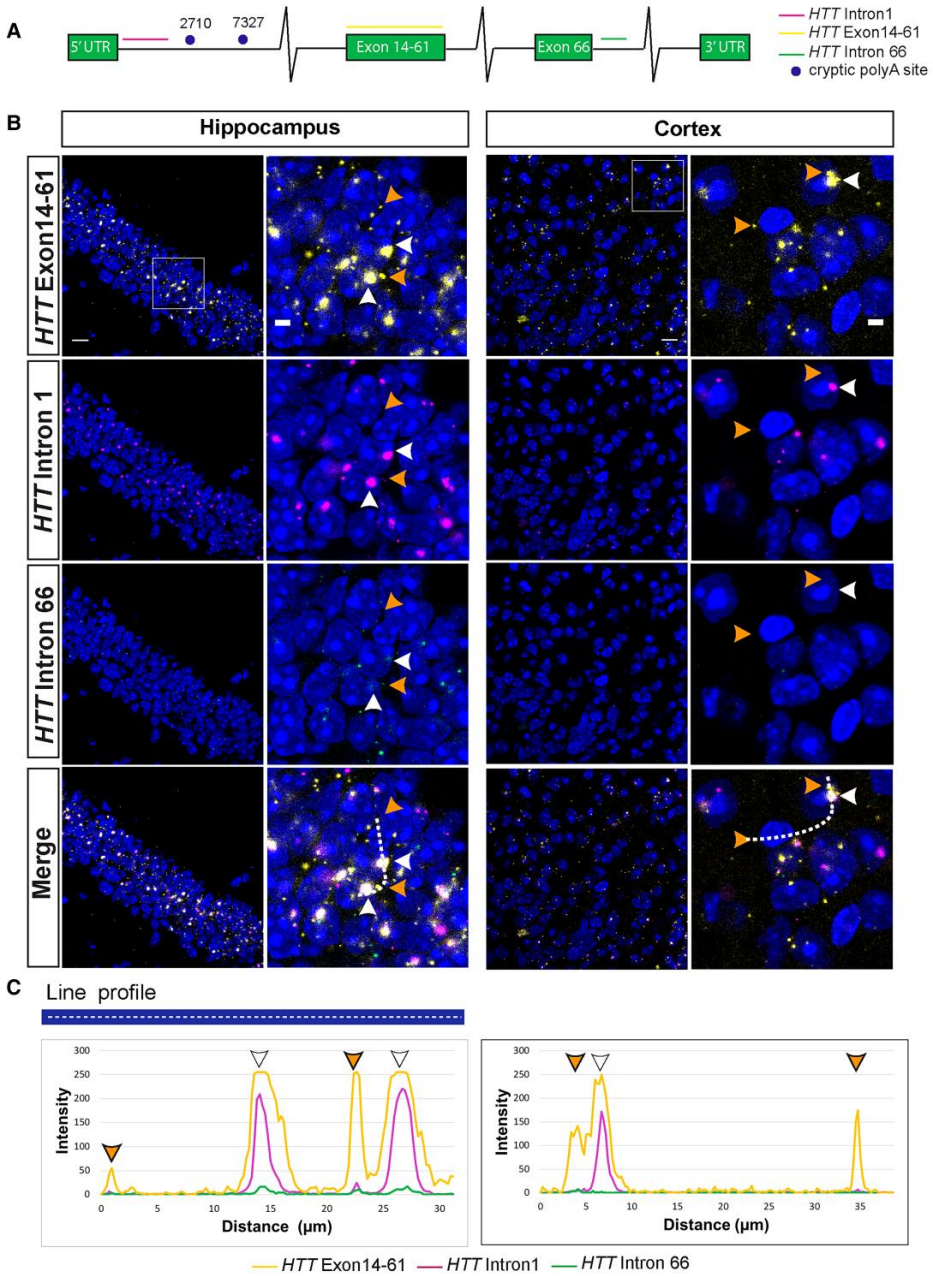
**Human *HTT* and *HTT1a* transcripts form nuclear RNA clusters in YAC128 brains.** (**A**) Schematic showing the location of the RNAscope probes on the human *HTT* transcript: full-length *HTT* (exons 14–61), *HTT1a* (*HTTintron1*) and preprocessed mRNA (*HTTintron66*). (**B**) Full-length *HTT* (yellow) and *HTT1a* (magenta) were frequently co-localized in nuclear RNA clusters in the hippocampus and cortex of YAC128 mice at 2 months of age (white arrowheads). Full-length *HTT* was also detected in the extranuclear space as single transcripts that did not co-localize with *HTT1a* (orange arrowheads). The *HTT1a* transcripts were predominantly detected in nuclear clusters. The *HTT intron 66* (green) probe visualized non-spliced pre-mRNA, which, although sparse, was present in both the nucleus and cytoplasm and did not co-localize with FL-*HTT* or *HTT1a*. (**C**) Intensity profiles of FL-*HTT*, *HTT1a* and *HTTintron66* signals. Peak intensities were recorded along the white dashed line shown in the merged image in **B**. The orange arrowheads indicate full-length human *HTT* outside of the nucleus, white arrowheads indicate human *HTT* and *HTT1a* that are co-localized in the nucleus. A statistical analysis of the transcript subcellular location is presented in Supplementary Fig. 1A and B. Nuclei were stained with DAPI (blue). The wild-type control sections at 2 months of age are shown in Supplementary Fig. 3A and B. YAC128 (*n* = 4). Scale bar = 20 μm in the main image and 5 μm in the cropped magnified image.

The qPCR and QuantiGene experiments demonstrated that the *HTT1a* transcript was present in YAC128 brains; however, these methods lacked detailed spatial and subcellular context. To visualize the location of the *HTT* transcripts, we took advantage of RNAscope technology, which allows the detection of a single RNA molecule with simultaneous background suppression. We designed probes to detect either human *HTT1a* (5′ sequences of intron 1), *FL-HTT* (exons 14–61) or unprocessed *HTT* pre*-*mRNA (intron 66) (Fig. 2A).

Cortical and hippocampal sections from YAC128 mice at 2 months of age were hybridized with the three RNAscope probes. Single *FL-HTT* transcripts were detected in the cytoplasm, consistent with the processed mRNA having been exported from the nucleus for translation (Fig. 2B and C). Surprisingly, the *FL-HTT* probe detected large RNA clusters in both hippocampal and cortical nuclei. The intron 1 probe identified a small number of cytoplasmic transcripts, as would be expected, given that the *HTT1a* transcript is translated to generate the exon 1 HTT protein.^7^ However, the vast majority of *HTT1a* was in the nucleus and co-localized with *FL*-*HTT* mRNA in the RNA clusters (Fig. 2B and C and Supplementary Fig. 1A and B). It is noteworthy that the *FL-HTT* mRNA in most of the RNA clusters, as well as single mRNAs present in the nucleus, appears to be spliced, as they were rarely detected by the intron 66 probe (Fig. 2B and C).

**Figure 3:**
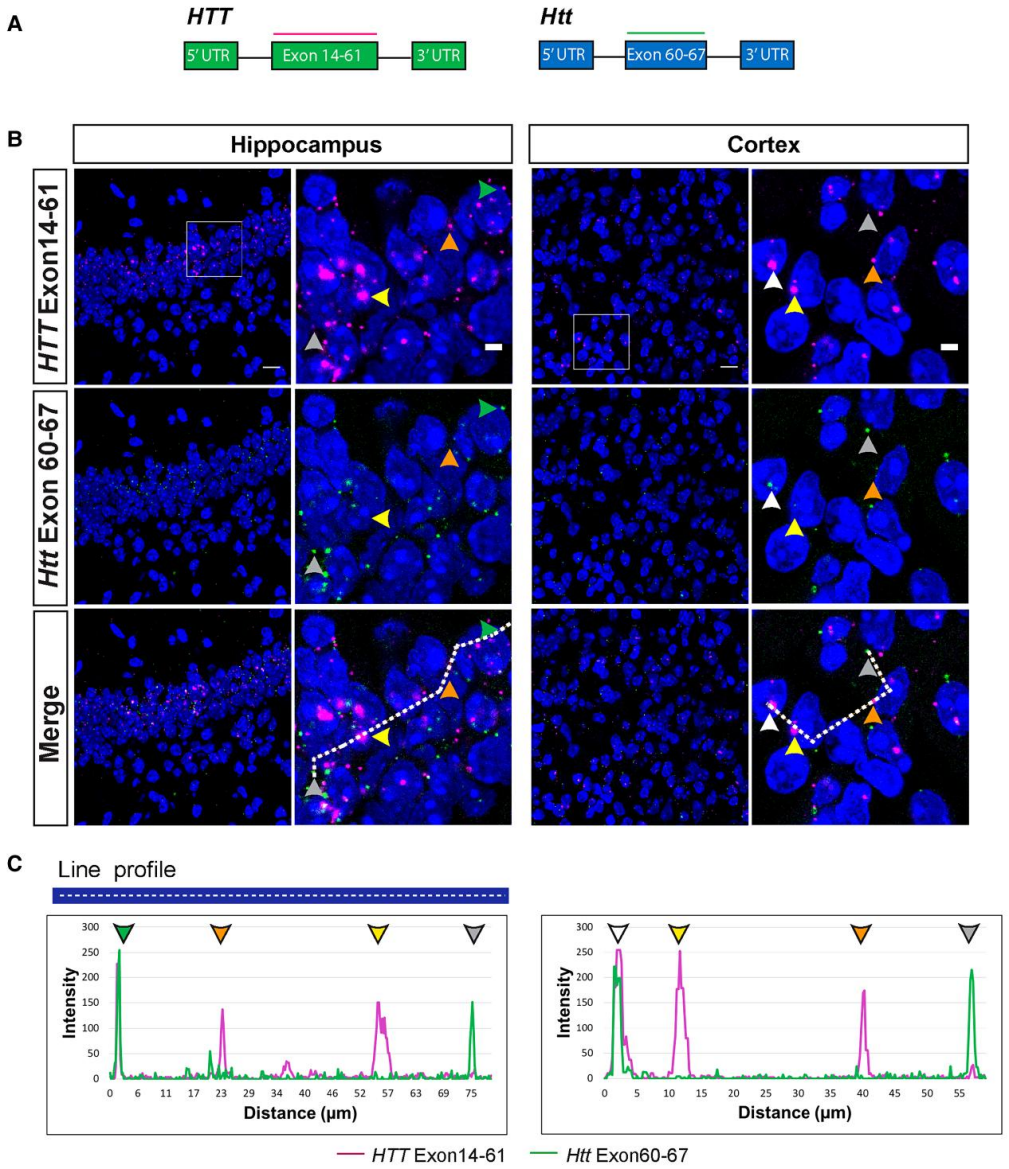
**Mouse *Htt* transcripts were present in the nucleus and cytoplasm, whereas human *HTT* mRNAs were retained in nuclei in YAC128 brains**. (**A**) Schematic location of the full-length human (exons 14–61) and mouse (exons 60–67) huntingtin RNAscope probes. (**B**) Full-length human *HTT* (magenta) was present in large nuclear clusters (yellow arrowheads) and as single transcripts in the extranuclear space (orange arrowheads). Full-length mouse *Htt* (green) was predominantly detected outside of the nucleus (grey arrowheads). Human and mouse huntingtin transcripts only rarely co-localized in both the nucleus (white arrowheads) and cytoplasm (green arrowheads). (**C**) Intensity profiles of full-length mouse and human huntingtin transcript signals. Peak intensities were recorded along the white dashed line shown in the merged image in panel (**B**). Yellow arrowheads indicate large nuclear clusters containing human *HTT*, orange arrowheads indicate human *HTT* transcripts outside of the nucleus, grey arrowheads indicate mouse *Htt* transcripts outside of the nucleus, white arrowheads indicate co-localization of human *HTT* and mouse *Htt* inside the nucleus and green arrowheads indicate co-localization of human *HTT* and mouse *Htt* in the cytoplasm. A statistical analysis of the transcript subcellular location is presented in Supplementary Fig. 1C and D. Nuclei were stained with DAPI (blue). The wild-type control sections from mice at 2 months of age are shown in Supplementary Fig 3A and B. YAC128 (*n* = 4). Scale bar = 20 μm in the main image and 5 μm is the cropped magnified image.

The specificity of the human *HTT* probes was confirmed, as they gave no signal when hybridized to brain sections from wild-type mice (Supplementary Fig. 2A). In contrast to human *HTT*, mouse wild-type *Htt* was detected as single mRNA molecules both in the nucleus and cytoplasm of hippocampal and cortical sections from YAC128 and wild-type mice (Fig. 3A and B and Supplementary Figs 2B and 4B). Occasionally, mouse *Htt* co-localized with the large human *HTT* clusters, suggesting these structures could potentially trap other mRNA molecules (Fig. 3B and C and Supplementary Fig 1C and D).

The nuclear retention of *HTT* transcripts was emphasized by the fact that most housekeeping mRNAs, including *Ubc*, *Ppib* and *Polar2a*, were primarily localized in the cytoplasm (Supplementary Fig. 3). To confirm that the *HTT* mRNA clusters were not an artefact of the RNAscope assay, but rather reflected a biological phenomenon, we designed and validated a probe that used a smaller number of ZZ-pairs for signal amplification (Supplementary Fig. 3A). This probe also recognized the *HTT* mRNA clusters trapped in nuclear structures (Supplementary Fig. 4B).

Next, we determined whether nuclear RNA clusters could also be detected in the brains of a knock-in mouse model of Huntington’s disease. RNAscope was performed on hippocampal and cortical sections from zQ175 mice, in which mouse exon 1 *Htt* has been replaced with a mutant version of human exon 1 *HTT*. The probes used were to full-length mouse *Htt* (*FL-Htt* exons 60–67), *Htt1a* (5′ sequences of mouse intron 1) or unprocessed *Htt* pre*-*mRNA (mouse intron 2) (Supplementary Fig. 5A). In contrast to the signals obtained in YAC128 mice, RNA clusters were not observed; both the *FL*-*Htt* and *Htt1a* probes detected single transcripts that were predominantly cytoplasmic (Supplementary Fig. 5B).

### Greatest levels of soluble exon 1 HTT and HTT aggregation were detected in the YAC128 cerebellum

**Figure 4:**
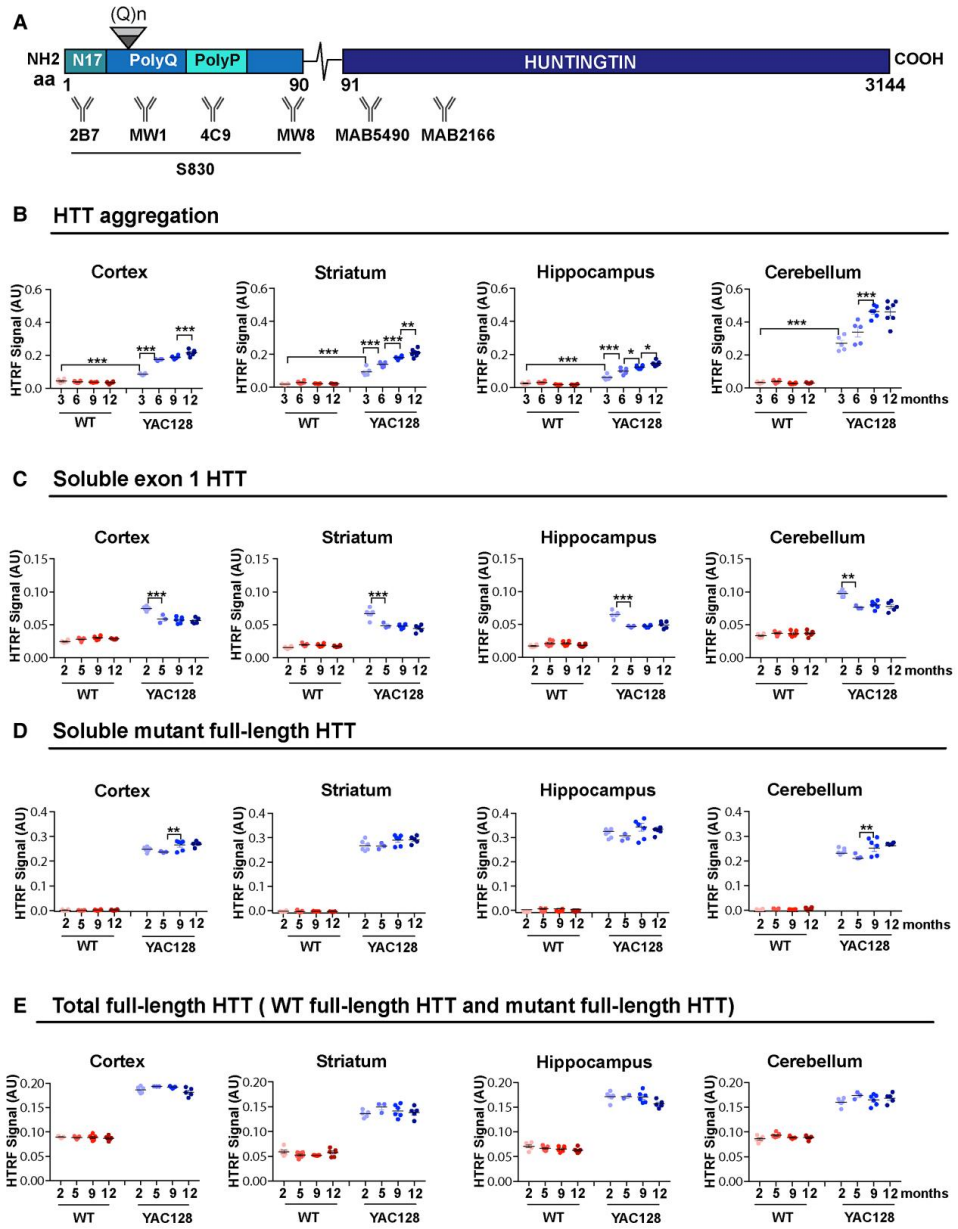
**HTRF assays detect greater levels of soluble exon 1 HTT and HTT aggregation in the cerebellum than in forebrain regions.** (**A**) Schematic indicating the position of the HTT epitopes detected by the antibodies used in the HTRF assays (Supplementary Table 3). S830 is a polyclonal sheep antibody that was raised against exon 1 HTT with 53Q. (**B**) HTT aggregation, as detected by the 4C9-MW8 assay, increased from 3 to 12 months of age in all brain regions. (**C**) Exon 1 mutant HTT (2B7-MW8) levels decreased between 2 and 5 months of age in all brain regions and then remained relatively stable. (**D**) Full-length mutant HTT (MW1-MAB5490) remained relatively stable up to 12 months of age in all brain regions. (**E**) The levels of total full-length HTT (MAB5490-MAB2166) were higher in the YAC128 mice, which contain three copies of the huntingtin gene. Samples for any given HTT assay were run on the same plate, and therefore the levels between brain regions can be compared. *n* = 6. Statistical analysis was one- or two-way ANOVA with Tukey’s *post hoc* correction. Error bars = mean ± SEM *P ≤ 0.05, **P ≤ 0.01, ***P ≤ 0.001. M = months; WT = wild-type; aa = amino acid.

We have recently established a series of HTRF assays to track changes in soluble and aggregated HTT isoforms in Huntington’s disease mouse tissues.^21^ Therefore, we applied these bioassays to investigate how soluble and aggregated HTT levels might change with disease progression in brain regions from YAC128 mice. HTRF relies on the detection of a fluorescent signal that is emitted when two antibodies recognizing specific HTT epitopes are in close proximity.^30^ The donor and receptor antibodies for the selected HTRF assays, respectively, were as follows: 4C9 and MW8 for aggregated HTT; 2B7 and MW8 for soluble mutant exon 1 HTT; MW1 and MAB5490 for soluble mutant full-length HTT; MAB5490 and MAB2166 for total full-length HTT (mutant and wild-type) (Fig. 4A).

**Figure 5:**
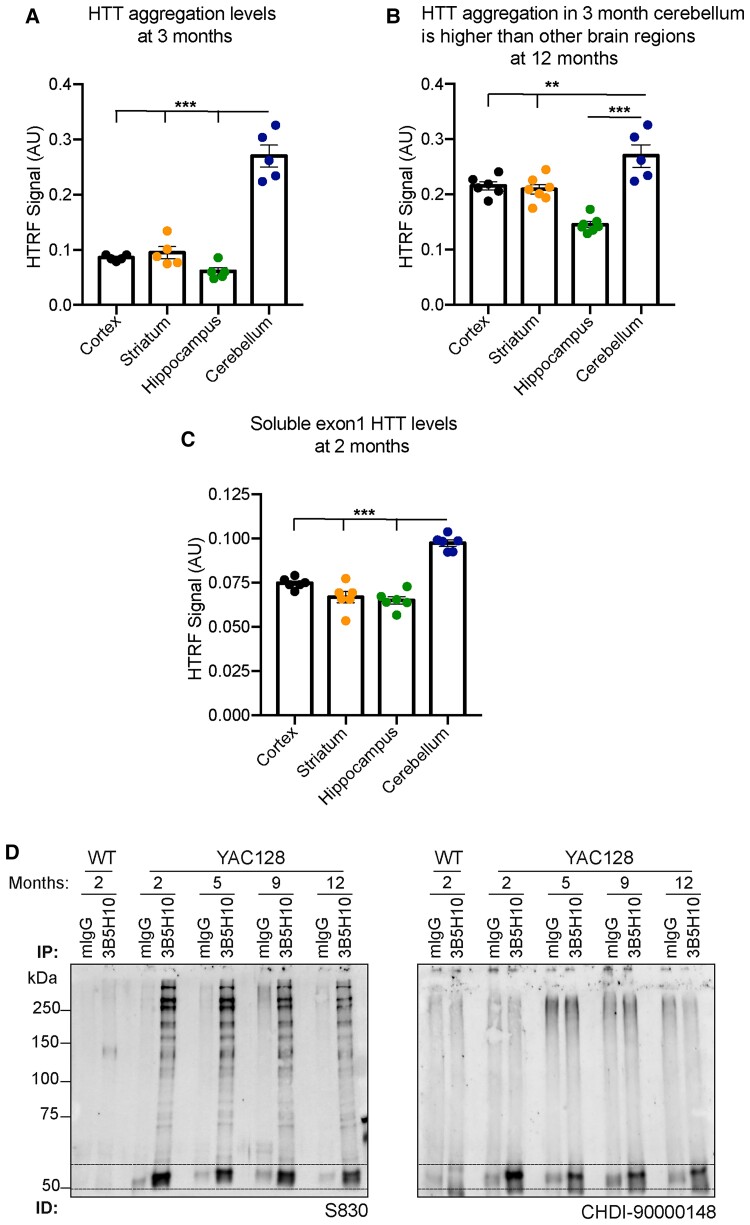
**Comparison of aggregated and soluble HTT isoforms between brain regions and with disease progression.** (**A**) HTT aggregation levels were greatest in the cerebellum at 3 months of age. (**B**) HTT aggregation levels were greater in the cerebellum at 3 months of age than in the cortex, striatum or hippocampus at 12 months. (**C**) Soluble exon 1 HTT levels were highest in the cerebellum (*n* = 6). (**D**) Huntingtin was immunoprecipitated with 3B5H10 from YAC128 cortical lysates at 2, 5, 9 and 12 months of age and wild-type lysates at 2 months. Western blots were immunoprobed with the S830 or CHDI-90000148 antibodies. Dotted lines indicate the location of the exon 1 HTT protein. Error bars = mean ± SEM. Statistical analysis was one-way ANOVA with Tukey’s *post hoc* correction. *P ≤ 0.05, **P ≤ 0.01, ***P ≤ 0.001.

The 4C9-MW8 assay is a well-established bioassay for detecting levels of aggregated HTT.^21,31^ Statistically significant levels of aggregation could be detected in all four brain regions at 3 months of age, which in all cases increased with disease progression (Fig. 4B). The greatest level of aggregation was detected in the cerebellum, with lower levels in the striatum, cortex and hippocampus (Fig. 5A). Interestingly, the level of aggregation in the cerebellum at 3 months of age was already at a level not reached by 12 months in the striatum and cortex (Fig. 5B).

The 2B7-MW8 and MW1-MAB5490 assays allow the levels of soluble exon 1 HTT and soluble full-length mutant HTT to be determined. The 2B7-MW8 assay is specific for the exon 1 HTT protein, as MW8 acts as a neo-epitope antibody for the C-terminus of exon 1 HTT.^21,26^ Remarkably, at 2 months of age, the level of exon 1 HTT was greatest in the cerebellum, with comparably lower levels in the cortex, striatum and hippocampus (Fig. 5C). The level of exon 1 HTT decreased from 2 to 5 months of age in all brain regions, after which it remained relatively stable (Figs 4C and 5D). The levels of soluble mutant full-length HTT (MW1-MAB5490) were stable over the course of the disease (Fig. 4D). Finally, total full-length HTT (MAB5490-MAB2166) levels were higher in YAC128 than wild-type mice, consistent with these mice having three copies of HTT (Fig. 4E).

### Nuclear huntingtin aggregates are present before 3 months of age in YAC128 brain regions

**Figure 6:**
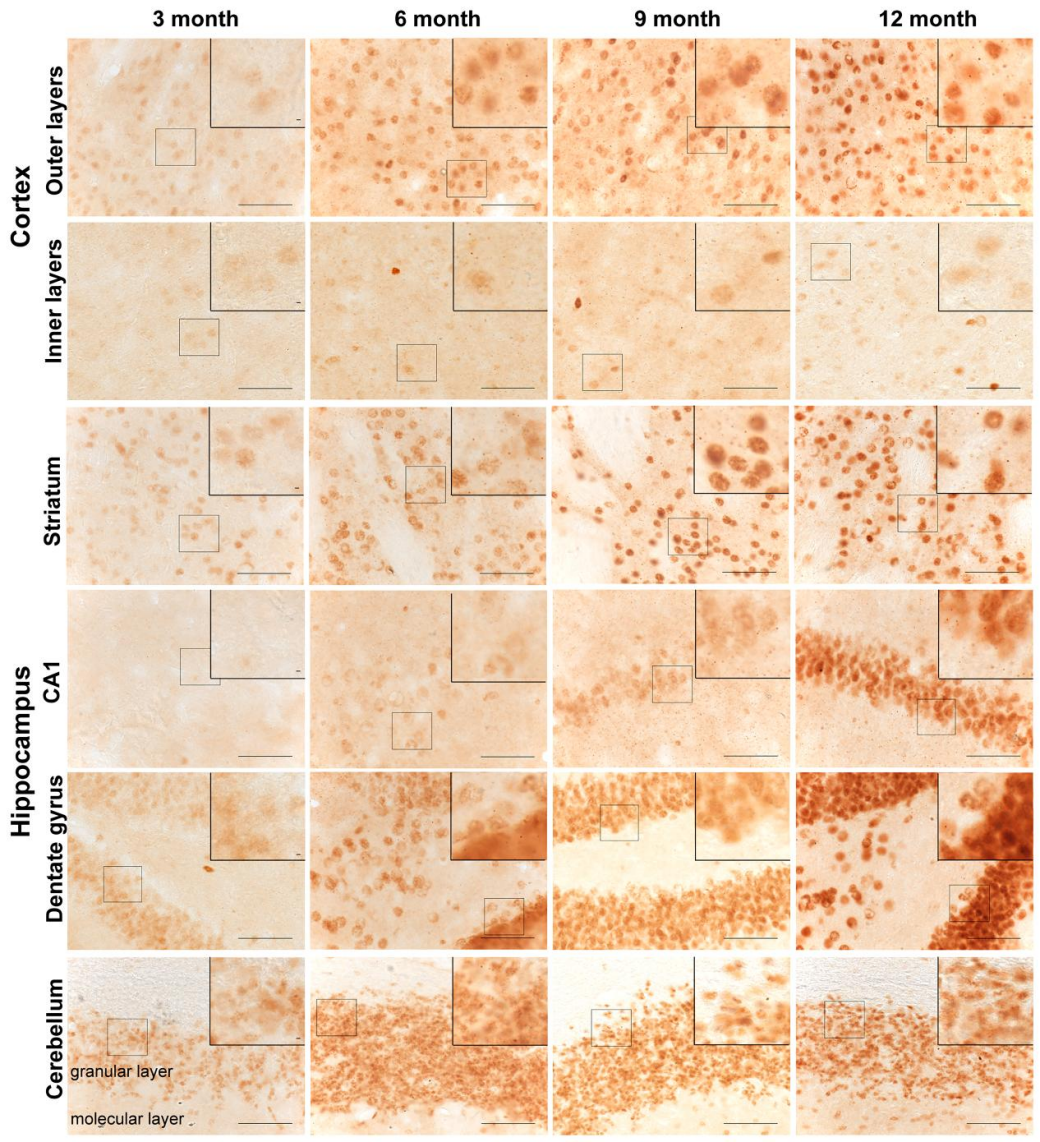
**Spatiotemporal appearance of HTT aggregation in YAC128 mice.** Coronal sections from 3-, 6-, 9- and 12-month-old YAC128 mice were immunostained with the S830 antibody to visualize HTT aggregates. A diffuse nuclear S830 immunostain, indicative of HTT aggregation, was readily identified in the striatum, outer cortical layers, dentate gyrus of the hippocampus and granular layer of the cerebellum at 3 months of age, but not in the CA1 region of the hippocampus until 9 months. This nuclear immunostain increased in intensity with disease progression and small nuclear inclusions were observed only rarely (see zoomed sections). By 6 months of age, extranuclear inclusions were readily apparent in the outer and inner layers of the cortex and the striatum and in the CA1 region of the hippocampus by 9 months. The location of the images from within the brain sections is illustrated in Supplementary Figs 6 and 7. The wild-type control sections from mice at 12 months of age are shown in Supplementary Fig. 8. YAC128 (*n* = 3), wild-type (*n* = 1). Scale bar = 20 μm.

We next performed immunohistochemistry to determine how the temporal and spatial appearance of HTT aggregates correlated with the progressive increase in HTT aggregation detected by HTRF. Coronal sections from YAC128 and wild-type mice at 3, 6, 9 and 12 months of age were immunostained with the S830 anti-HTT antibody, which readily detects aggregated HTT. At 3 months of age, aggregated HTT, in the form of a diffuse nuclear stain, was apparent in the outer layers of the cortex, the striatum and the dentate gyrus of the hippocampus, and this increased in intensity during the course of the disease (Fig. 6 and Supplementary Figs 6A, C and 7B). While the diffuse nuclear stain was apparent in the inner layers of the cortex in 3-month-old YAC128 mice, it remained relatively sparse in these layers up to 12 months (Fig. 6 and Supplementary Fig. 6B). Interestingly, and in keeping with the HTRF data, the cerebellum displayed a relatively high intensity of S830 staining at 3 months, which was most evident in the granular layer, with the molecular layer becoming increasingly affected over the 12-month period (Fig. 6 and Supplementary Fig. 7C). This S830 diffuse nuclear immunostain was not detected in the CA1 subfield of the hippocampus until 9 months (Fig. 6 and Supplementary Fig. 7A).

The HTT aggregation in nuclei remained diffuse and small nuclear inclusions were only rarely apparent, for example, in the striatum at 12 months of age (Fig. 6). High-power images revealed that cytoplasmic inclusions were present in the outer and inner cortical layers and the striatum by 6 months and the CA1 subfield of the hippocampus by 9 months, and these increased in density with age (Fig. 6). They were comparatively rare in the dentate gyrus and cerebellum (Fig. 6). Importantly, wild-type sections showed no detectable S830 staining at any age under investigation (Supplementary Fig. 8).

### YAC128 mice gained weight but did not develop progressive motor phenotypic deficits

A range of behavioural phenotypes have been reported in YAC128 mice but with considerable variability between laboratories in the degree of impairment detected.^17,32–34^ Therefore, we set out to assess longitudinal behavioural phenotypes in YAC128 mice using our standard battery of tests. Progressive changes in body weight, grip strength, rotarod performance and activity measures were recorded in YAC128 males (*n* = 11) and females (*n* = 10) and their wild-type littermate males (*n* = 9) and females (*n* = 10) monthly from 2 to 12 months of age.

Consistent with previous data^32,33^ both YAC128 males and females gained weight faster than their wild-type counterparts (Supplementary Fig. 9A). Body weight is known to influence rotarod performance and locomotor activity^35^; therefore, we introduced weight as a covariate when analysing the grip strength, rotarod and activity data. There was no difference in the change in hind- and fore-limb grip strength between YAC128 and wild-type mice up to 12 months (Supplementary Fig. 9B). To assess changes in balance and coordination, mice were tested for their ability to walk on an accelerating rotarod. Rotarod performance changed little with age for both YAC128 and wild-type mice, and when weight differences were taken into consideration, there was no difference in the change in rotarod performance between YAC128 and wild-type mice over the 12-month period (Supplementary Fig. 9C). Exploratory activity was assessed in an open field arena for a period of 30 min. The total distance travelled decreased for both YAC128 and wild-type mice between 2 and 12 months of age. When differences in weight were taken into consideration, there were no consistent differences in the total distance travelled between wild-type and YAC128 mice (Supplementary Fig. 9D).

### YAC128 MEFs: a tool for screening human *HTT* and *HTT1a* lowering agents

**Figure 7:**
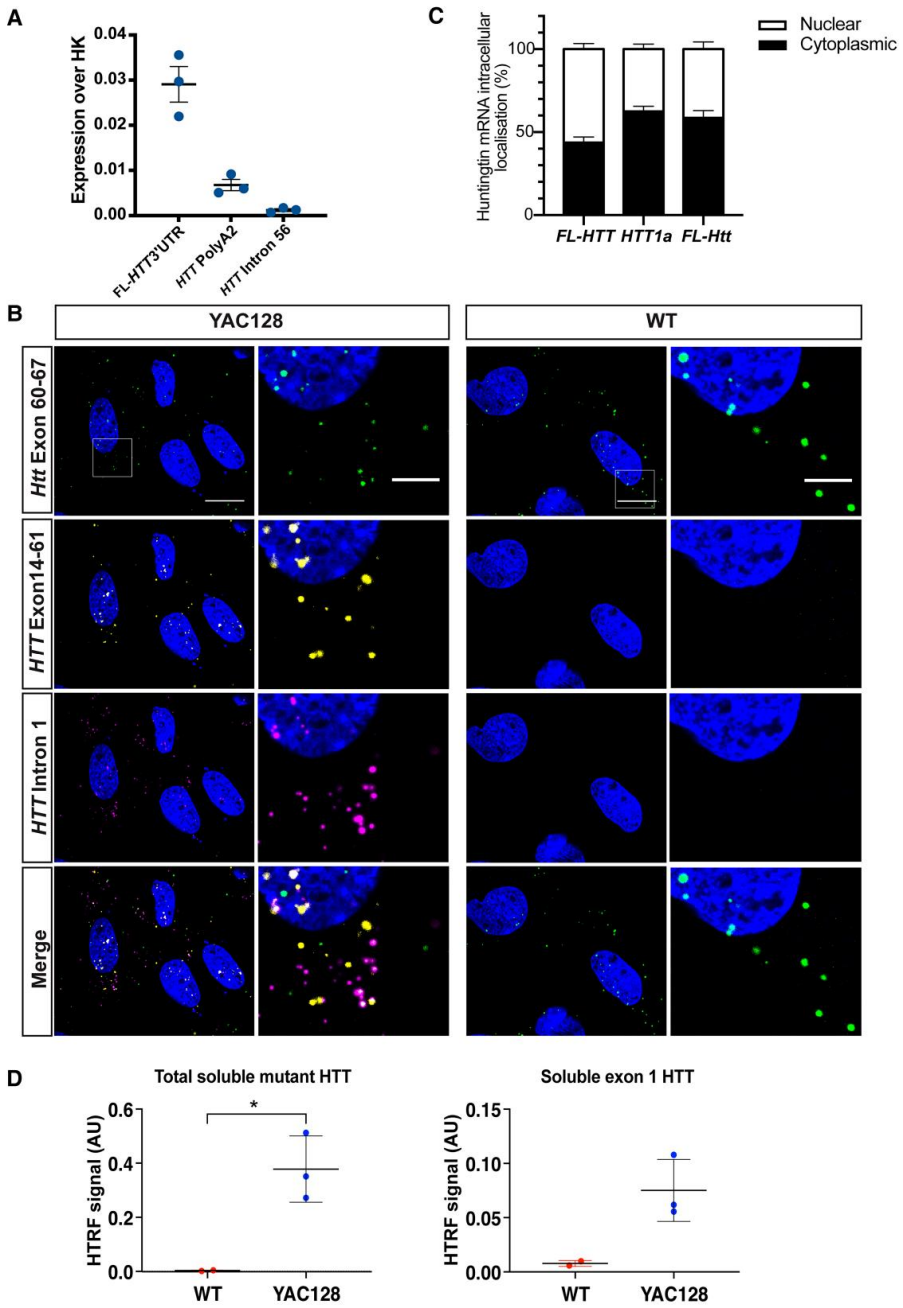
***HTT1a* is present in MEFs from YAC128 mice.** Quantitative PCR analysis revealed that (**A**) *HTT1a* (*HTT* PolyA2) *FL-HTT* (FL*-HTT* 3′ UTR) were present in the YAC128 MEFs (*n* = 3 biological replicates). (**B**) Percentage of nuclear and cytoplastic localization of different huntingtin transcripts. One-way ANOVA of percentage of transcripts in the nucleus, full-length human *HTT* compared to *HTT1a* (*P* = 0.002), full-length human *HTT* compared to mouse *Htt* (*P* = 0.02). (**C**) RNAscope on YAC128 MEFs revealed that wild-type mouse *FL-Htt* (green), human *FL-HTT* (yellow) and *HTT1a* (magenta) were present as single transcripts in the cytoplasm. Human *FL-HTT* and *HTT1a* were more likely to be found in the nucleus than mouse *FL-Htt*, where they frequently co-localized (*n* = 3 biological replicates). Scale bar = 20 μm in the main image and 5 μm in the magnified image. (**D**) Total soluble mutant HTT (2B7-MW1) and exon 1 HTT (2B7-MW8) could be detected in the YAC128 MEF lines (*n* = 3 biological replicates) by HTRF and not in wild-type lines (*n* = 2 biological replicates). YAC128 line 7.1 showed the highest huntingtin expression at both the RNA and protein levels. Student’s *t*-test, *P ≤ 0.05. Error bars = mean ± SEM. WT = wild type; HK = housekeeping genes.

We have previously shown that MEFs, derived from the zQ175 knock-in mice, constitute a reliable cellular model to study incomplete splicing events.^20^ To create a system that would allow screening of agents targeting human *HTT* transcripts, we generated transformed MEF lines from YAC128 mice (*n* = 3). We then used qPCR to show that both the *HTT1a* and *FL-HTT* (Fig. 7A) transcripts could be detected in the three YAC128 MEFs. Our RNAscope analysis had revealed that *FL-HTT* and *HTT1a* mRNAs were retained as nuclear RNA clusters in YAC128 brains (Fig. 2B and C). These clusters may have implications for the ability of potential therapeutic agents to target *HTT* transcripts and, therefore, we used RNAscope to assess the intracellular distribution of *HTT* transcripts in the YAC128 MEFs. Consistent with our *in vivo* data, housekeeping mRNAs were predominantly cytoplasmic with some single transcripts present in the nucleus (Supplementary Fig. 10A). Similarly, mouse *FL-Htt* mRNA signals were mostly detected in the cytoplasm (Fig. 7C), in accordance with previously published data for primary mouse fibroblasts.^36^ Although the large RNA clusters detected in brain were not apparent, the human *FL-HTT* and *HTT1a* mRNAs were observed to co-localize in the nucleus (Fig. 7B). However, quantification found no evidence of increased levels of human *HTT1a* in the nucleus as compared to mouse *FL-Htt* (Fig. 7C). Consistent with our *in vivo* data, the nuclear *FL-HTT* transcripts were mostly spliced, as they were rarely detected with the intron 66 RNAscope probe (Supplementary Fig. 11A). Next, we used HTRF assays to demonstrate that the wild-type and mutant soluble HTT isoforms could be detected in the three YAC lines. Levels of total soluble mutant HTT and exon 1 HTT were present in the YAC128 and not the wild-type MEFs (Fig. 7D).

**Figure 8:**
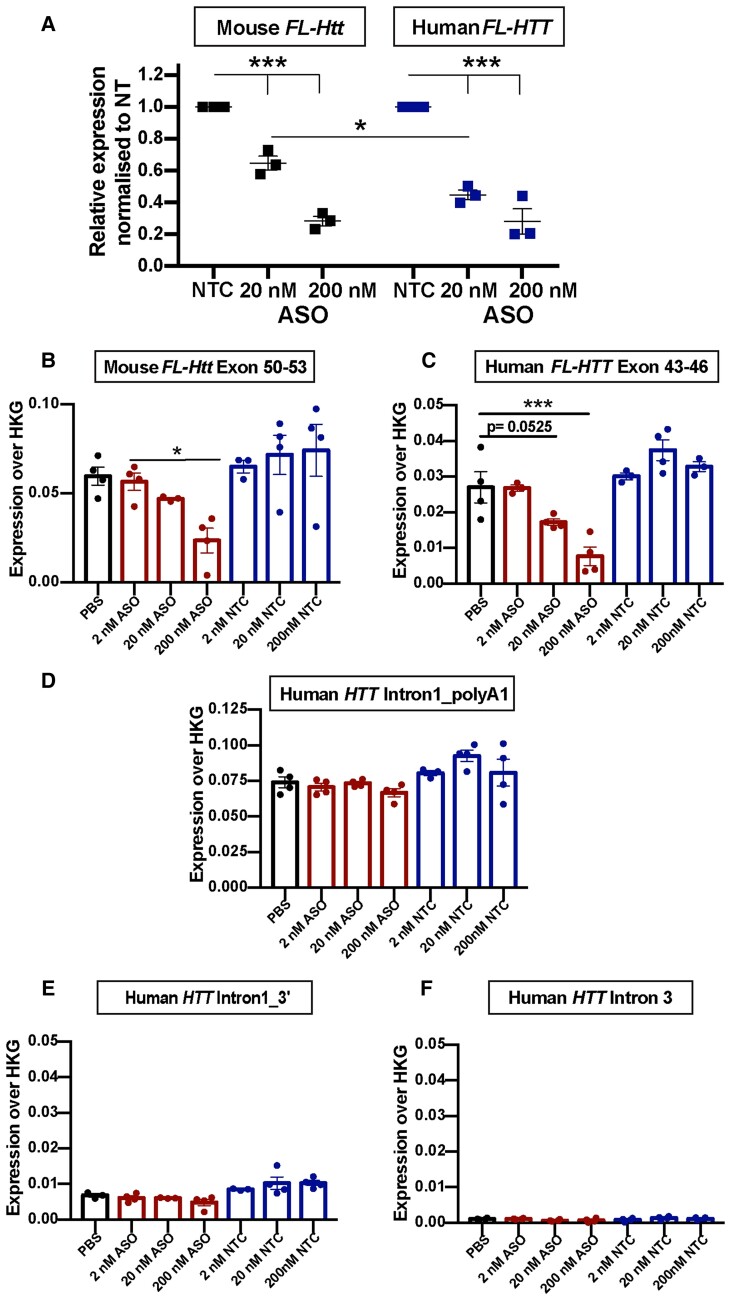
**YAC128 MEFs provide a tool for screening agents designed to lower *HTT* transcript levels.** (**A**) YAC128 MEFs were transfected with ASOs targeting the human *FL-HTT* and mouse *FL-Htt* transcripts or with a non-targeting control (NTC). Quantitative PCR analysis showed that 20 nM ASO was more efficient in lowering human *FL-HTT* levels than mouse *FL-Htt*, whereas 200 nM ASO decreased *FL-HTT* and *FL-Htt* to the same extent, 48 h post transfection. *n* = 3 biological replicates/genotype. Error bars = mean ± SEM. Statistical analysis was two-way ANOVA with Tukey’s *post hoc* correction for the effect on huntingtin lowering and Bonferroni *post hoc* correction for the effect between human and mouse huntingtin. (**B**–**F**) YAC128 MEFs were transfected with either PBS, the *FL-HTT*/*FL-Htt* targeting ASO or non-targeting control (NTC). The 15-plex QuantiGene assays was used to measure the levels of (**B**) mouse *FL-Htt*, (**C**) human *FL-HTT*, (**D**) human *HTT1a* (human intron 1_polyA1), human intron 1 3′ and (**F**) intron 3. *n* = 3–4 technical replicates of YAC128 MEF-7.1. Error bars = mean ± SEM. Statistical analysis was one-way ANOVA with Dunnet’s *post hoc* correction. *P = 0.014, ***P = 0.001.

To determine whether the YAC128-MEFs could be used to assess the effect of *HTT*-lowering agents, we transfected one of the YAC128 MEF lines with 20 or 200 nM *HTT*-ASO, which targets exon 42 of the *FL-HTT* transcript, or with a non-targeting control (NTC) ASO. The *HTT*-ASO degrades the *HTT* mRNA by an RNase H1-dependent mechanism.^37^ The level of human *FL-HTT* and mouse *FL-Htt* knockdown was assessed by qPCR. Transfection of 20 nM *HTT*-ASO resulted in a greater decrease (*P* ≤ 0.05) in human *FL-HTT* (55% ± 3%) than mouse *FL-Htt* (35% ± 4%) with a 200 nM dose resulting in comparable further reductions in human *FL-HTT* (72% ± 7%) and mouse *FL-Htt* (72% ± 3%) as compared to the NTC ASO (Fig. 8A).

Finally, we sought to determine whether our QuantiGene multiplex panel could be used to assess the effects of *HTT-*lowering agents directly from cell lysates. One of the YAC128 MEF lines (MEF-7) was transfected with 2, 20 or 200 nM of the *HTT*-ASO or of the NTC ASO. Cells were lysed and the QuantiGene 15-plex panel was used to measure mouse *FL-Htt*, human *FL-HTT*, human *HTT* intronic sequences and the levels of five mouse housekeeping genes (Supplementary Table 2). The comparative decrease in levels of mouse *FL-Htt* (Fig. 8B) and human *FL-HTT* (Fig. 8C) in response to treatment with the *HTT*-ASO was comparable to that determined by qPCR (Fig. 8A), and the level of *HTT1a* was unchanged (Fig. 8D).

## Discussion

In the presence of an expanded CAG repeat, aberrant processing of the huntingtin mRNA generates the *HTT1a* transcript, which encodes the aggregation-prone and pathogenic exon 1 HTT protein.^7^ This occurs in post-mortem brains and fibroblast cultures from Huntington’s disease patients^8^ and has been shown to correlate with disease progression and HTT aggregate deposition in knock-in mouse models.^29^ Here we demonstrated that human *HTT1a* is produced throughout the brain in YAC128 mice, that are transgenic for the entire human *HTT* gene. We found that both full-length *HTT* and *HTT1a* were retained in the nucleus as RNA clusters as well as being present in the cytoplasm as single transcripts. *HTT1a* encodes the pathogenic exon 1 HTT protein, which was present throughout the YAC128 brain, a level that correlated with HTT aggregation in specific brain regions. These results have important implications for the design of agents aimed at lowering the levels of pathogenic forms of huntingtin.

We used both qPCR and QuantiGene analysis to show that the mutant human *HTT* mRNA in YAC128 mice was alternatively processed to generate the *HTT1a* transcript and found that the human *FL-HTT* and *HTT1a* transcripts accumulated as nuclear RNA clusters in the brains of YAC128 mice. The retention of mRNAs in nuclear ‘foci’ has been described for a range of tandem tri-, tetra- and penta-nucleotide repeat disorders including myotonic dystrophy types 1 and 2, fragile X tremor ataxia syndrome, spinocerebellar types 8, 10 and 31 and Huntington's disease like-2.^38^ RNA foci in myotonic dystrophy type 1 caused by an expanded CUG repeat in the 3′ UTR of the *DMPK* mRNA,^39^ co-localized with muscleblind-like splicing regulator 1 (MBNL1)^40^ and were located at the periphery of nuclear speckles.^39,41^ This phenomenon has been previously reported in Huntington’s disease patient-derived fibroblasts as well as lymphoblasts,^42^ in which ‘foci’ also co-localized with MBNL1 and were associated with the splicing speckle marker SC35.^42^ The similarities between these diseases would suggest that the formation of RNA foci is a consequence of mRNA structures formed by the expanded tandem tri-, tetra- and penta-nucleotide repetitive sequences. However, we did not observe nuclear RNA clusters in the brains of zQ175 knock-in mice, which contain a chimeric protein between human exon 1 *HTT* and mouse *HTT* and harbour expansions of approximately 200 CAGs. The disease-specific differences in the huntingtin transcripts between YAC128 and zQ175 are the intron 1 sequences in *HTT1a*, which in YAC128 mice are human, extending for 7327 bp, and in zQ175 are mouse, extending for 1145 bp. The secondary and tertiary mRNA structures of the human *HTT1a* mRNA may drive cluster formation, possibly through a phase separation process involving the sequence-specific gelation of RNAs.^43^

The formation of RNA ‘foci’ is widely regarded as being an RNA toxicity-related phenomenon, and in the case of myotonic dystrophy, MBNL1 depletion, through sequestration into RNA foci, causes the aberrant splicing of genes linked to a wide range of clinical symptoms.^44^ However, in the case of Huntington’s disease, these nuclear clusters might exert a protective effect, by retaining *HTT1a* in the nucleus and preventing it from being translated to generate the highly aggregation-prone and pathogenic exon 1 HTT protein. We applied our array of HTRF assays^21^ to monitor changes in the levels of soluble HTT isoforms and aggregated HTT in YAC128 mice over the course of the disease up to 12 months of age. At 2 months, the exon 1 HTT protein could be detected in all four brain regions studied: cortex, striatum, hippocampus and cerebellum (Fig. 5). The highest exon 1 HTT levels correlated with the greatest aggregation signal, supporting the hypothesis that the mutant exon 1 HTT protein initiates the aggregation process.

We performed immunohistochemistry to visualize the temporal–spatial appearance of aggregated HTT in YAC128 brains. We have previously shown that the diffuse/granular appearance of mutant HTT in cell nuclei, as detected by the S830 antibody, represents an aggregated form of HTT.^25,26^ Aggregation of HTT in the nucleus first appears as this diffuse/granular stain that fills the entire nucleus; and subsequently, as disease progresses, this may coalesce to form nuclear inclusions, as in the case of R6/2 mice with ∼200 CAGs, or alternatively remain relatively diffuse over the entire course of the disease, as for R6/2 mice with 90 CAGs.^25^ Our observation that aggregated HTT in the nucleus also first appears as a diffuse staining pattern in YAC128 mice is consistent with previous studies.^34,45,46^ We found that aggregated HTT had been deposited in the outer layers of the cortex, the striatum, the dentate gyrus of the hippocampus as well as in the granular layer of the cerebellum by 3 months of age, in keeping with our HTRF data. This appearance of HTT aggregation is earlier than previously reported for YAC128 mice on a C57BL/6J background,^45,46^ a discrepancy most likely due to experimental procedures. The widespread distribution of aggregated HTT is in keeping with Bayram-Weston *et al*.,^46^ who first detected aggregation in the amygdala, thalamus, cerebellum, hippocampus, piriform cortex and ventral striatum.^46^ Our data are consistent with all previous studies, in that only small and very sparse nuclear inclusions could be detected by 12 months of age, if at all,^17,46–48^ and that cytoplasmic inclusions were also comparatively sparse.^46^ This pattern of aggregation is remarkably similar to that in R6/2 mice with 90 CAGs.^25^ Interestingly, the reduction in soluble exon 1 HTT in R6/2 (CAG)_90_ mice was also similar to that in YAC128, with a large reduction occurring at a young age after which levels were relatively stable.^25^ If exon 1 HTT is driving aggregation in the YAC128 mice, the level of exon 1 HTT with 125 glutamines leads to a similar pattern of aggregation as occurs with the level of exon 1 HTT with 90 glutamines in R6/2 mice, but over a more protracted timescale.

Given that widespread HTT aggregation occurs before 3 months of age, we set out to determine when we could first detect motor-phenotypes in YAC128 using our standard battery of behavioural and phenotypic tests. As previously reported, we found that YAC128 mice on a C57BL/6J background gained weight with age,^32,33^ a consequence of having an extra copy of full-length HTT.^49^ As body weight can modulate the performance in behavioural tasks,^35^ we used weight as a covariate in our statistical analyses. Consistent with Menalled *et al.*,^32^ who performed a detailed analysis of YAC128 mice on both an FVB/N and C57BL/6J background, we found no evidence for a rotarod impairment, reduction in grip strength or changes in open field behaviour up to 12 months of age. However, YAC128 mice on a C57BL/6J background have been shown to display deficits in other motor^50^ and cognitive tacks.^50,51^

We had previously shown that *HTT1a* can be readily detected in MEFs derived from zQ175 mice and that a QuantiGene multiplex assay could be used to screen approaches to lower full-length mouse *Htt* and *Htt1a* levels.^20^ Therefore, we established SV40 T-antigen-transformed MEF lines from YAC128 embryos and showed that *HTT1a* could be detected by qPCR, although the large nuclear RNA clusters present in YAC128 brains were not apparent. We used a well-characterized ASO targeting full-length huntingtin to test the utility of the human *HTT* QuantiGene multiplex assay as a tool for screening cell lysates. This simultaneously allowed the detection of the human *FL-HTT*, *HTT1a* and mouse *FL-Htt* transcripts as well as intronic and reference gene controls. It provides a rapid means of comparing transcript levels between control samples and those that have been genetically modified, or treated with agents designed, to modulate huntingtin transcript levels. We found that while full-length mouse *Htt* and human *HTT* levels were decreased, the level of *HTT1a* remained unchanged. The YAC128 MEFs allow agents to be screened against the entire human gene, before further investigation in a system in which the effects on RNA clusters can be determined.

Our work demonstrates that human *HTT* undergoes incomplete splicing in YAC128 mice to produce the *HTT1a* transcript and that this mRNA is either retained in the nucleus in RNA clusters or exported to the cytoplasm and translated to produce the exon 1 HTT protein. Importantly, these RNA clusters containing full-length *HTT* and *HTT1a* have also been identified in both Huntington's disease post-mortem brains and mouse models in a related study (Ly *et al.,* in press). Moreover, we show that *HTT1a* is readily detected in YAC128-derived MEFs and that oligonucleotide-based compounds targeting *HTT* are efficient in lowering *HTT* levels in this model system. This makes YAC128-derived MEFs particularly useful as a screening tool to evaluate experimental therapies directly targeting the human *HTT* transcripts and to study the molecular underpinnings of incomplete splicing in the context of the human *HTT* gene. Our data have profound implications for the development of *HTT* lowering therapies. It is essential that agents that lower the levels of *HTT1a* are developed. These agents would have the advantage that they specifically target the exon 1 HTT protein, a form of mutant HTT that is known to be highly pathogenic, while leaving levels of the wild-type HTT unchanged. This is an important consideration as the degree to which wild-type HTT can be lowered is currently unknown.^52^ It remains to be established whether RNA clusters act as mediators of RNA-induced toxicity or contrarily they detain abnormal mRNA and thus curb the production of otherwise noxious mutant huntingtin proteins. Whether HTT targeting approaches will be efficient in dissolving RNA clusters, and what the biological consequences of that might be, is an essential question that needs to be elucidated.

## Supplementary Material

awac241_Supplementary_Data

## Abbreviations

3′ UTR: 3′ untranslated region
ASO: antisense oligonucleotide
HTRF: homogeneous time-resolved fluorescence
MEF: mouse embryonic fibroblast
YAC: yeast artificial chromosome
