# Supplementary material for: Alternative processing of human HTT mRNA with implications for Huntington’s disease therapeutics
Source: Brain. 2022 Jul 6;145(12):4409–24. doi: 10.1093/brain/awac241 (PMC9762945; doi:10.1093/brain/awac241)
Supplement: awac241_Supplementary_Data [file awac241_supplementary_data.pdf]

SUPPLEMENTARY MATERIAL

Alternative processing of human *HTT* mRNA with implications for Huntington's disease therapeutics.

Sandra Fienko[1], Christian Landles[1], Kirupa Sathasivam[1], Sean J. McAteer[1], Rebecca E. Milton[1], Georgina F. Osborne[1], Edward J. Smith[1], Samuel T. Jones[1], Marie K. Bondulich, Emily C. E. Danby[1], Jemima Phillips[1], Bridget A. Taxy[1], Holly B. Kordasiewicz[2], Gillian P. Bates[1].

[1]Department of Neurodegenerative Disease, Huntington's Disease Centre, and UK Dementia Research Institute at UCL, Queen Square Institute of Neurology, UCL, London, WC1N 3BG, UK

[2]Ionis Pharmaceuticals, Carlsbad, CA 92008, USA

Correspondence to: Gillian Bates

Department of Neurodegenerative Disease, Queen Square Institute of Neurology, UCL, Queen Square House, Queen Square, London WC1N 3BG, UK.

E-mail: gillian.bates@ucl.ac.uk

Supplementary Figure 1

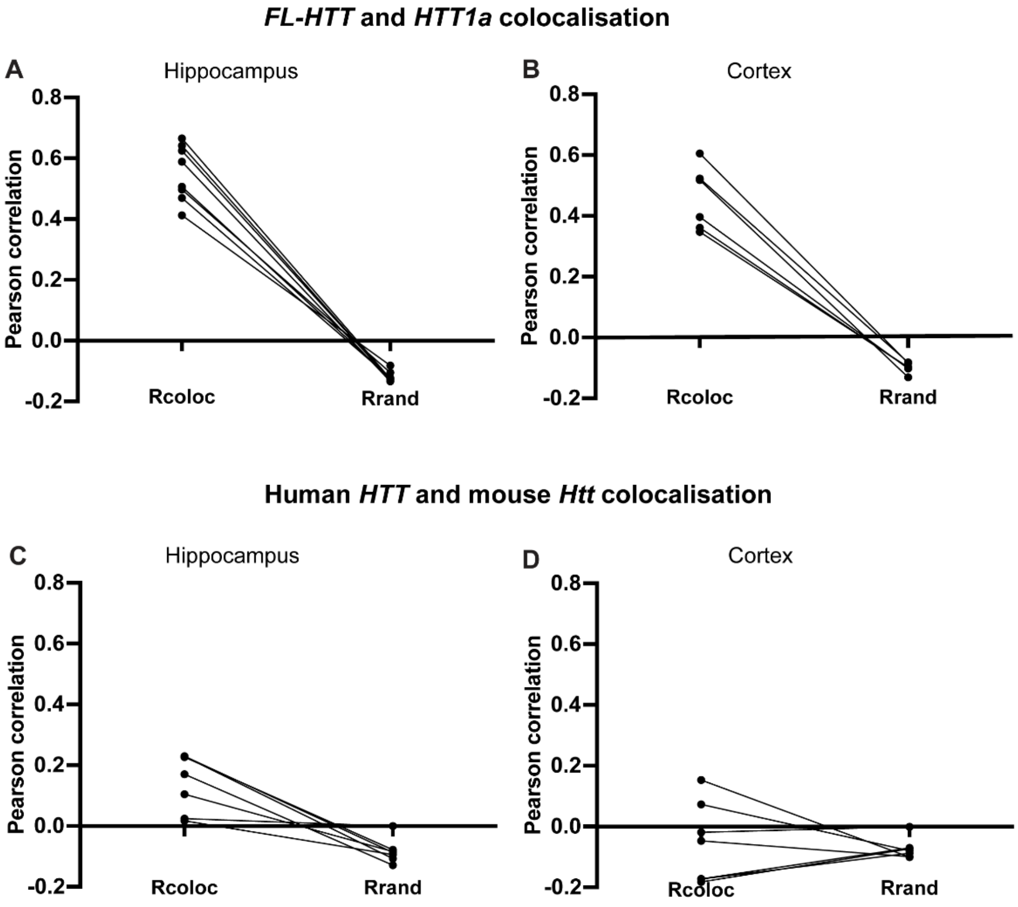

Supplementary Figure 1. Colocalisation of confocal images.
(**A, B**) Pearson correlation coefficients (R) calculated using the full-length human *HTT* and *HTT1a* images in (**A**) hippocampus and (**B**) cortex and for human HTT and mouse Htt in (**C**) the hippocampus and (**D**) cortex. The R value for colocalization (Rcoloc) is compared to the R value when the pixels have been randomly reshuffled (Rrand). The lines link the R values for the colocalised and reshuffled pixels for a single image. The human *HTT* and *HTT1a* transcripts were much more likely to colocalise than the human *HTT* and mouse *Htt* transcripts. 7 – 8 images were analysed for each brain region.

Supplementary Figure 2

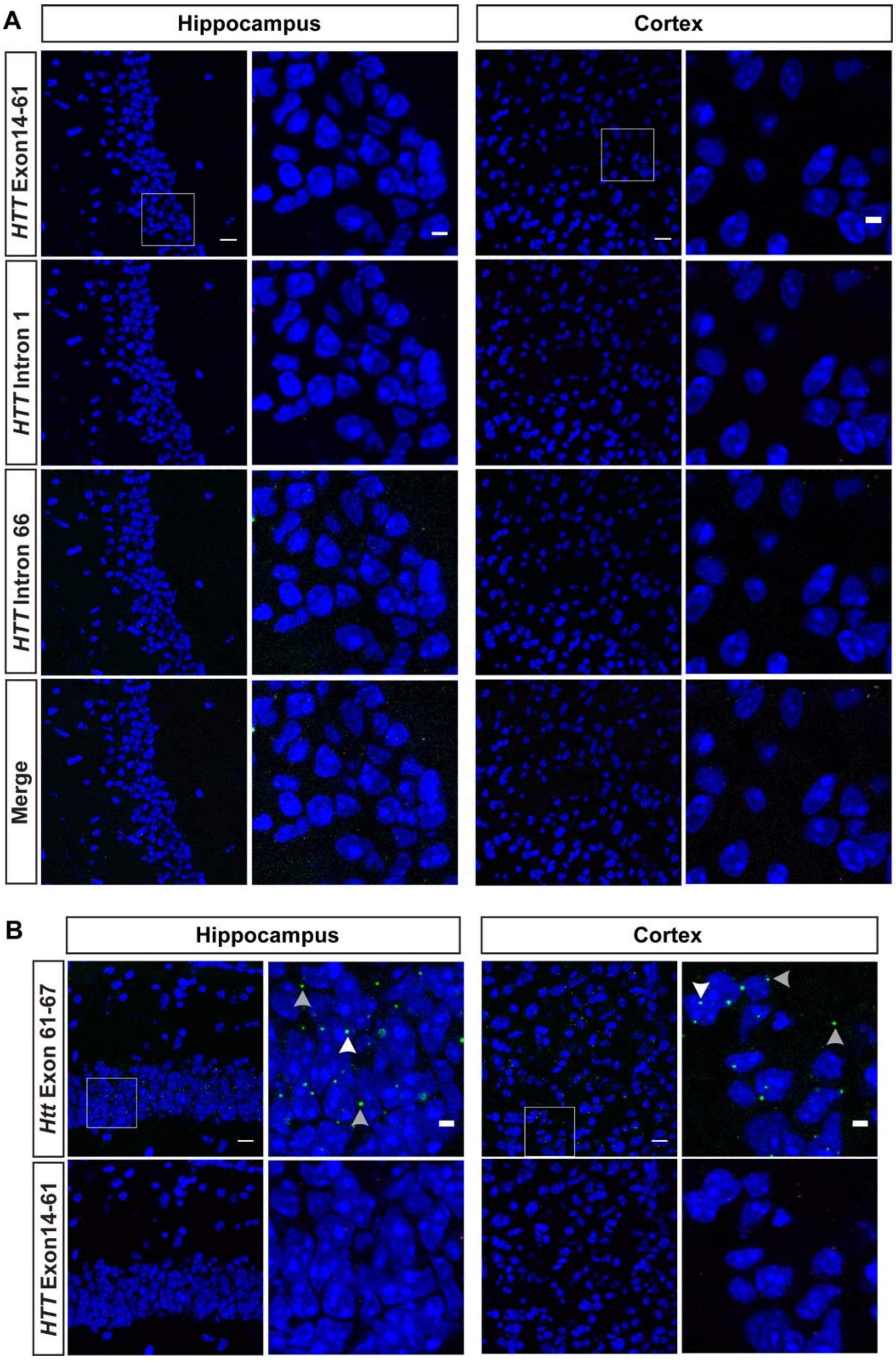

Supplementary Figure 2. **RNAscope probes to human *HTT* do not detect transcripts in the brains of wild-type mice.**

(A) Sagittal sections from wild-type brains at 2 months of age were hybridised with *FL-HTT*, *HTTintron1* and *HTTintron66* probes. Wild-type sections showed no staining with any of the human *HTT* probes. **(B)** Sagittal sections from wild-type animals were hybridised with RNAscope probes against human and mouse full-length huntingtin transcripts. Sections show no staining for FL-*HTT* (human), whereas FL-*Htt* (mouse) is predominantly cytoplasmic (grey arrowheads) with occasional signals detected in the nucleus (white arrowheads). Nuclei were stained with DAPI (blue). Wild type (n = 3). Scale bar = 20 μm in the main image (left-hand side) and 5 μm in the cropped magnified image (right-hand side).

Supplementary Figure 3

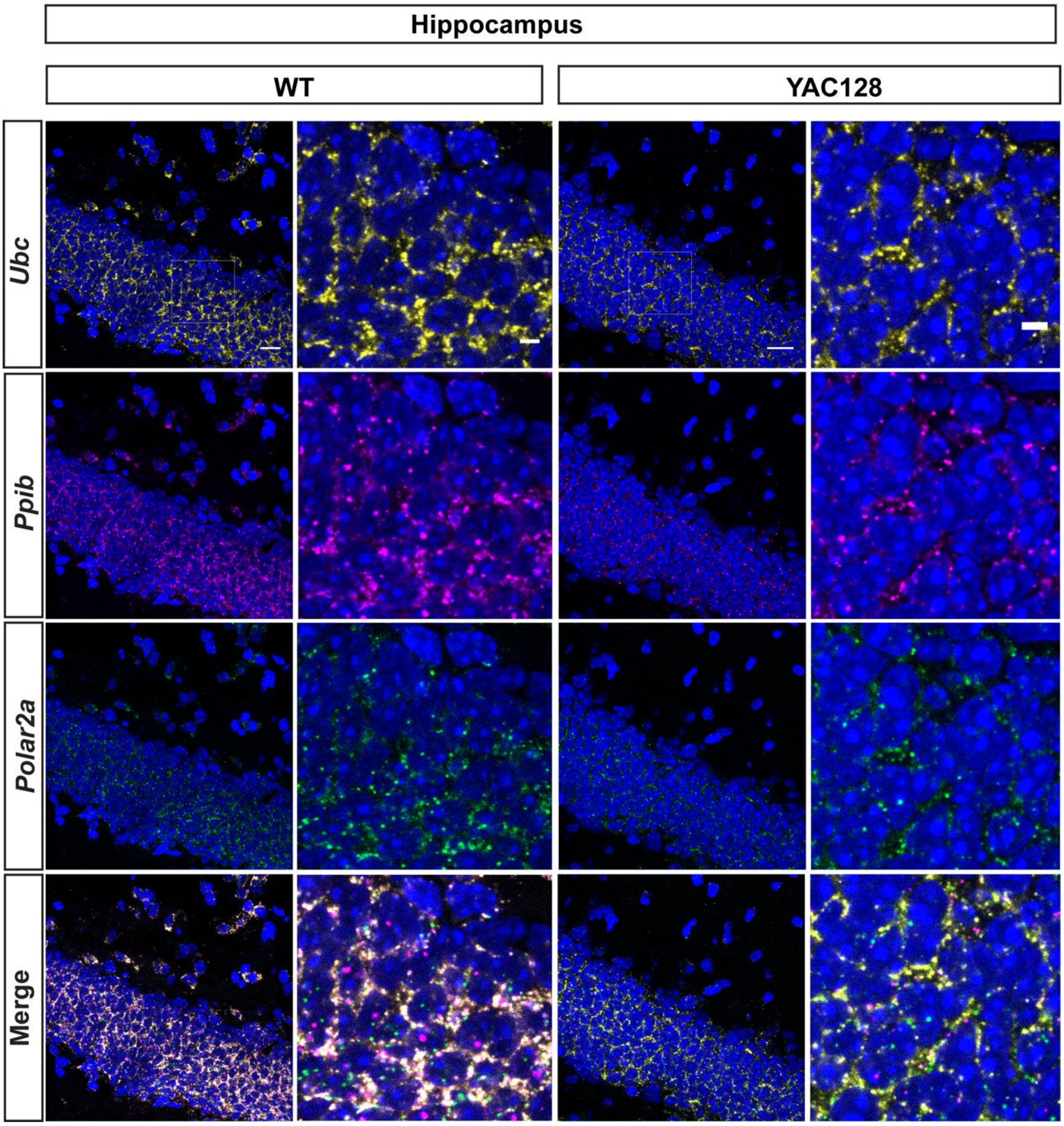

Supplementary Figure 3. Detection of mRNA transcripts for three differentially expressed housekeeping genes in the brains of the wild-type and YAC128 mice.
Sagittal sections from wild-type and YAC128 animals at 2 months of age were stained with a set of probes recognising housekeeping mRNAs. *Ubc* is a highly expressed transcript, *Ppib* is expressed at high to medium levels whereas *Polar2a* is a low abundant mRNA. Note that most of the transcripts reside outside of the nucleus. Nuclei were stained with DAPI (blue). YAC128 (n = 4), WT (n = 3). Scale bar = 20 µm in the main image (left-hand side) and 5 µm in the cropped magnified image (right-hand side). WT = wild type.

Supplementary Figure 4

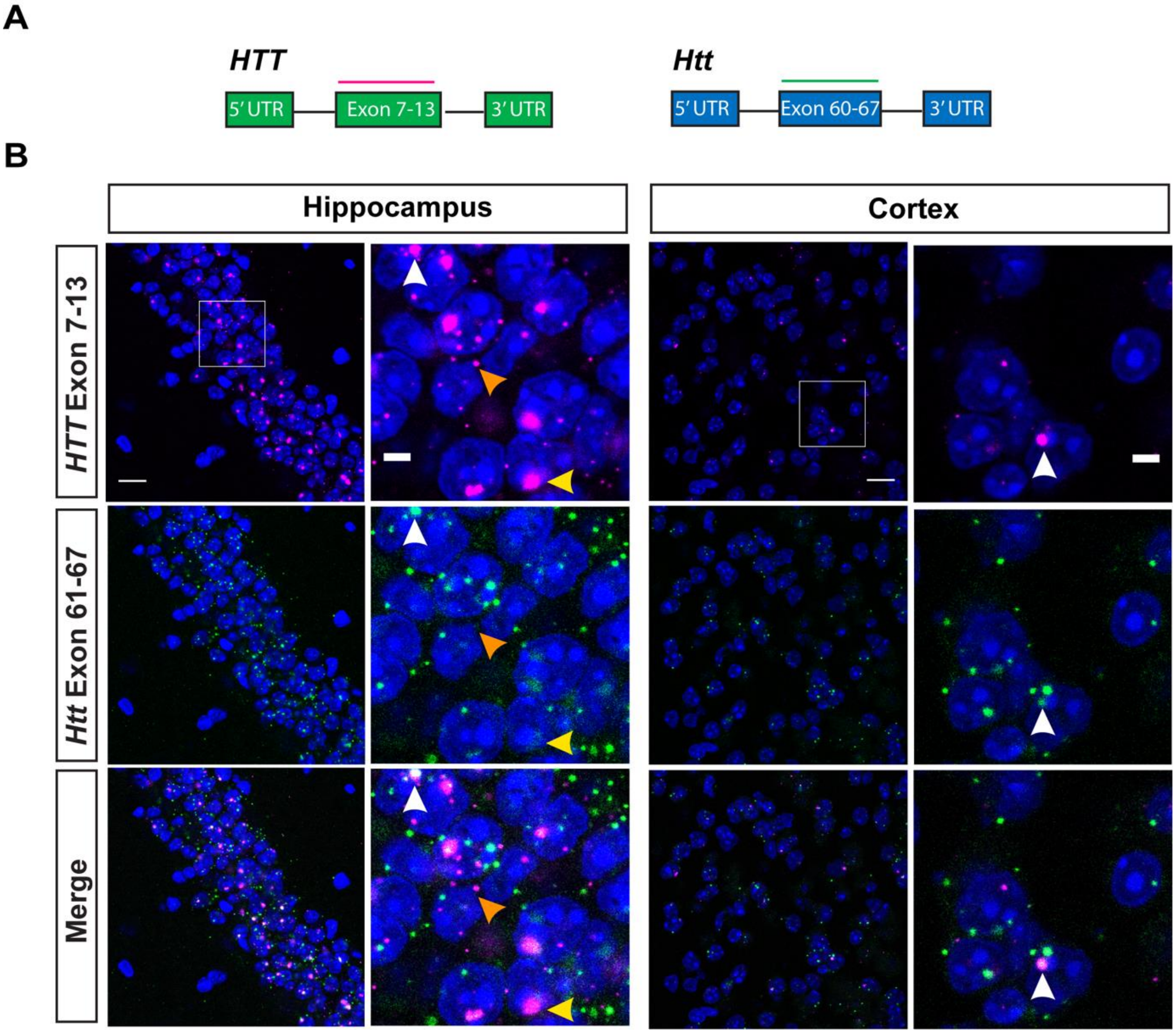

Supplementary Figure 4. An RNAscope probe to human *HTT* with a smaller ZZ number detected nuclear RNA clusters, while mouse *Htt* was predominantly cytoplasmic in the YAC128 brains.

(A) Schematic location of the full-length human and mouse huntingtin RNAscope probes. **(B)** Human *HTT* visualised with an RNAscope probe containing a lower ZZ number was localised to nuclear clusters (yellow arrowheads) and detected single transcripts in the extranuclear space (orange arrowheads). Mouse *Htt* was predominantly detected outside of the nucleus, however at times it was trapped within the nuclear RNA clusters (white arrowheads). Nuclei were stained with DAPI (blue). YAC128 (n = 4). Scale bar = 20 μm in the main image (left-hand side) and 5 μm in the cropped magnified image (right-hand side).

Supplementary Figure 5

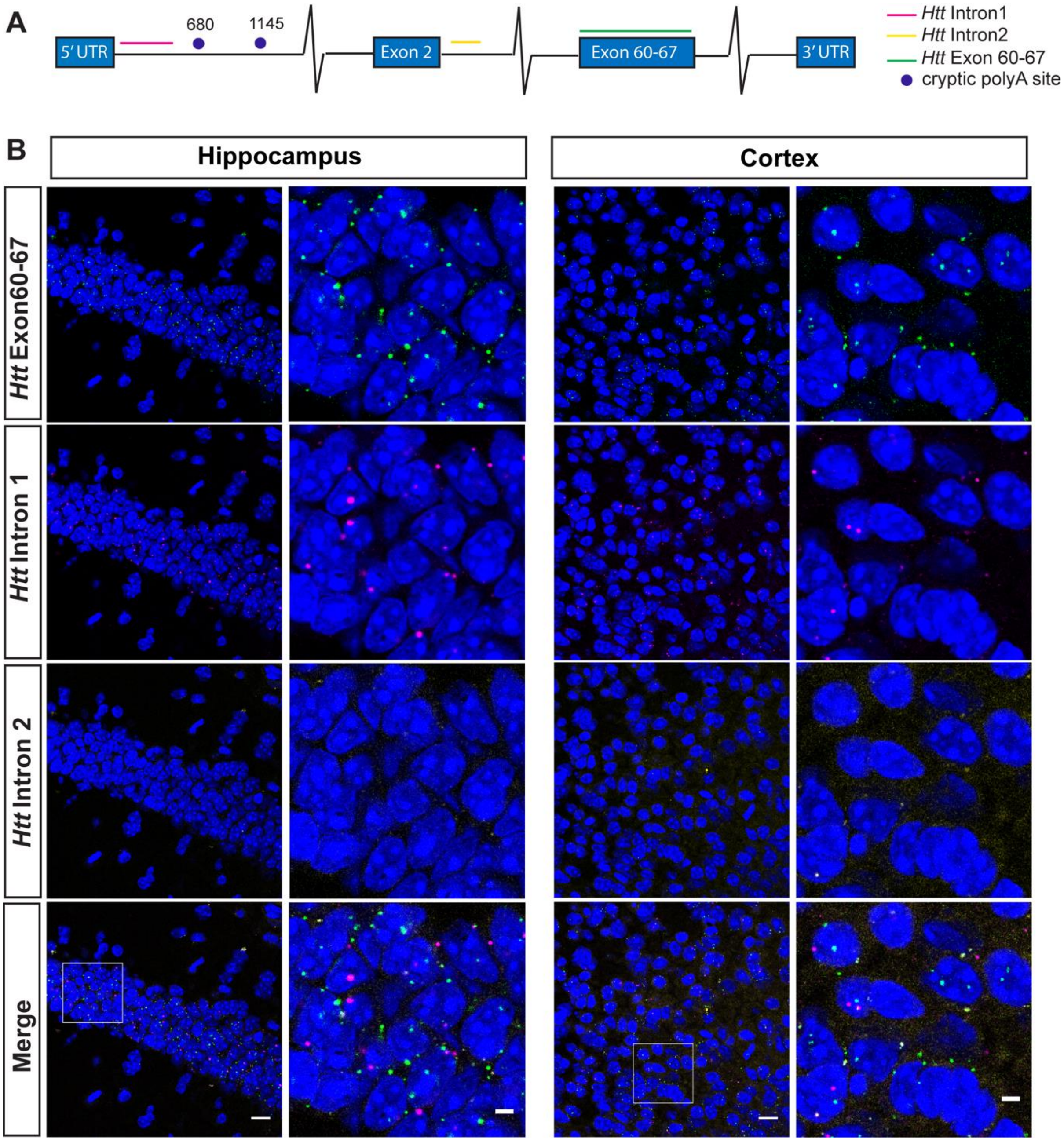

Supplementary Figure 5. **Mutant *Htt* does not form nuclei RNA clusters in the brains of zQ175 mice.**

(A) Schematic location of the RNAscope probes to mouse full-length *Htt* (exons 66-67), *Htt1a* (*Httintron1*) and pre-processed mRNA (*Httintron2*). **(B)** Sagittal sections from zQ175 animals at 2 months of age were hybridised with the *FL-Htt*, *Httintron1* and *Httintron2* probes. Both the FL-*Htt* and *Httintron1* probes detected transcripts that were predominantly cytoplasmic with occasional signals occurring in the nucleus. The *Httintron2* probe found no evidence of pre-processed *Htt* mRNA. Nuclei were stained with DAPI (blue). zQ175 (n = 2). Scale bar = 20 µm in the main image (left-hand side) and 5 µm in the cropped magnified image (right-hand side).

Supplementary Figure 6

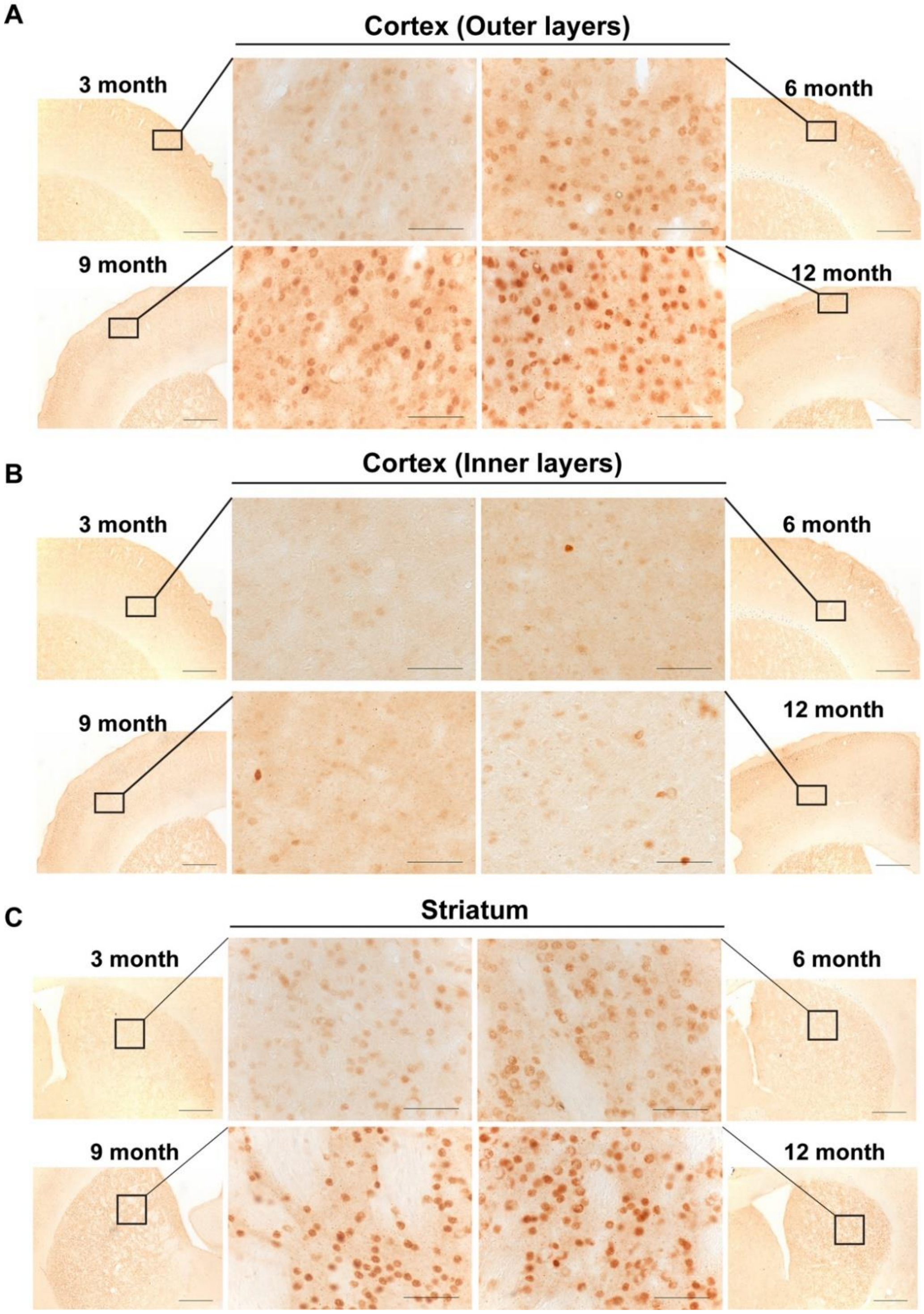

Supplementary Figure 6. Huntingtin aggregation in YAC128 brains.
The pattern of HTT aggregation in coronal sections from YAC128 brains at 3, 6, 9 and 12 months of age immunostained with S830 is shown for **(A)** outer cortical layers **(B)** inner cortical layers and **(C)** striatum. The outer thumbnails indicate the location of the images illustrated in Fig 5. YAC128 (*n* = 3). Centre panels, scale bar = 20 μm; thumbnail images, scale bar = 200 μm.

Supplementary Figure 7

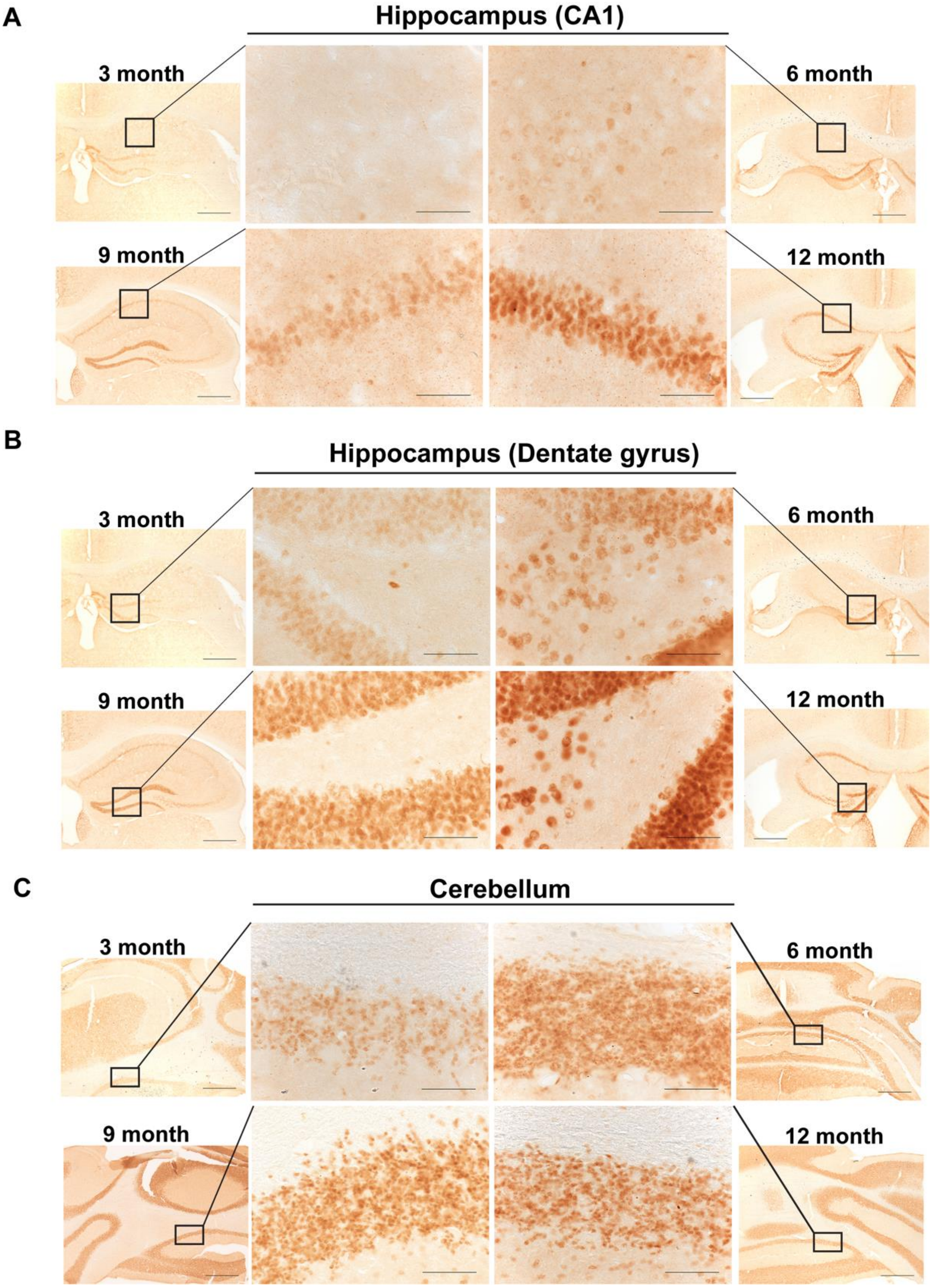

Supplementary Figure 7. Huntingtin aggregation in YAC128 brains.
The pattern of HTT aggregation in coronal sections from YAC128 brains at 3, 6, 9 and 12 months of age immunostained with S830 is shown for **(A)** CA1 hippocampal region **(B)** dentate gyrus and **(C)** cerebellum. The outer thumbnails indicate the location of the images illustrated in Fig 5. YAC128 (*n* = 3). Centre panels, scale bar = 20 µm; thumbnail images, scale bar = 200 µm.

Supplementary Figure 8

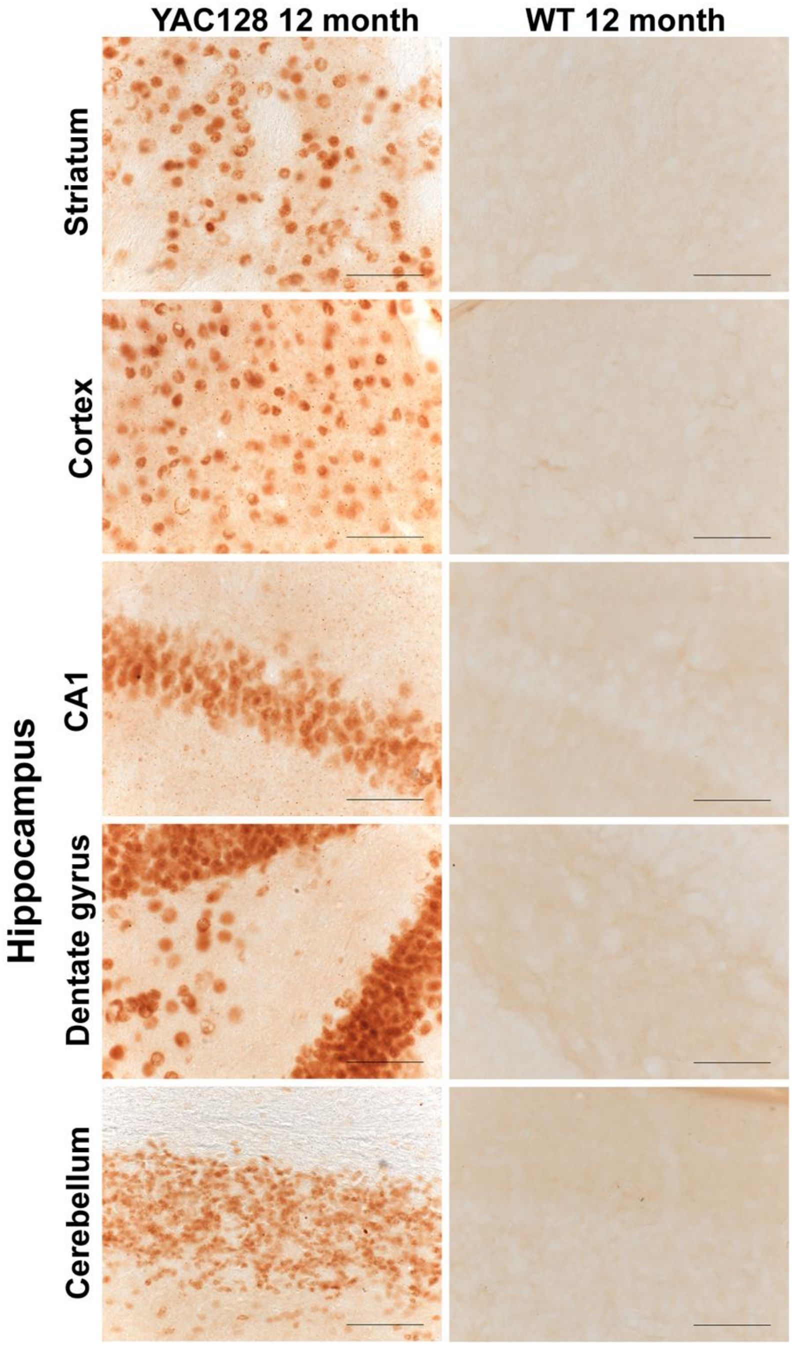

Supplementary Figure 8. S830 immunostaining was specific for huntingtin aggregation in YAC128 brains.

Coronal sections from YAC128 and wild-type brains at 12 months of age were immunostained with S830. WT sections showed no staining with the S830 antibody. YAC128 (n = 3), WT (*n* = 1). Scale bar = 20 μm. WT = wild type.

Supplementary Figure 9

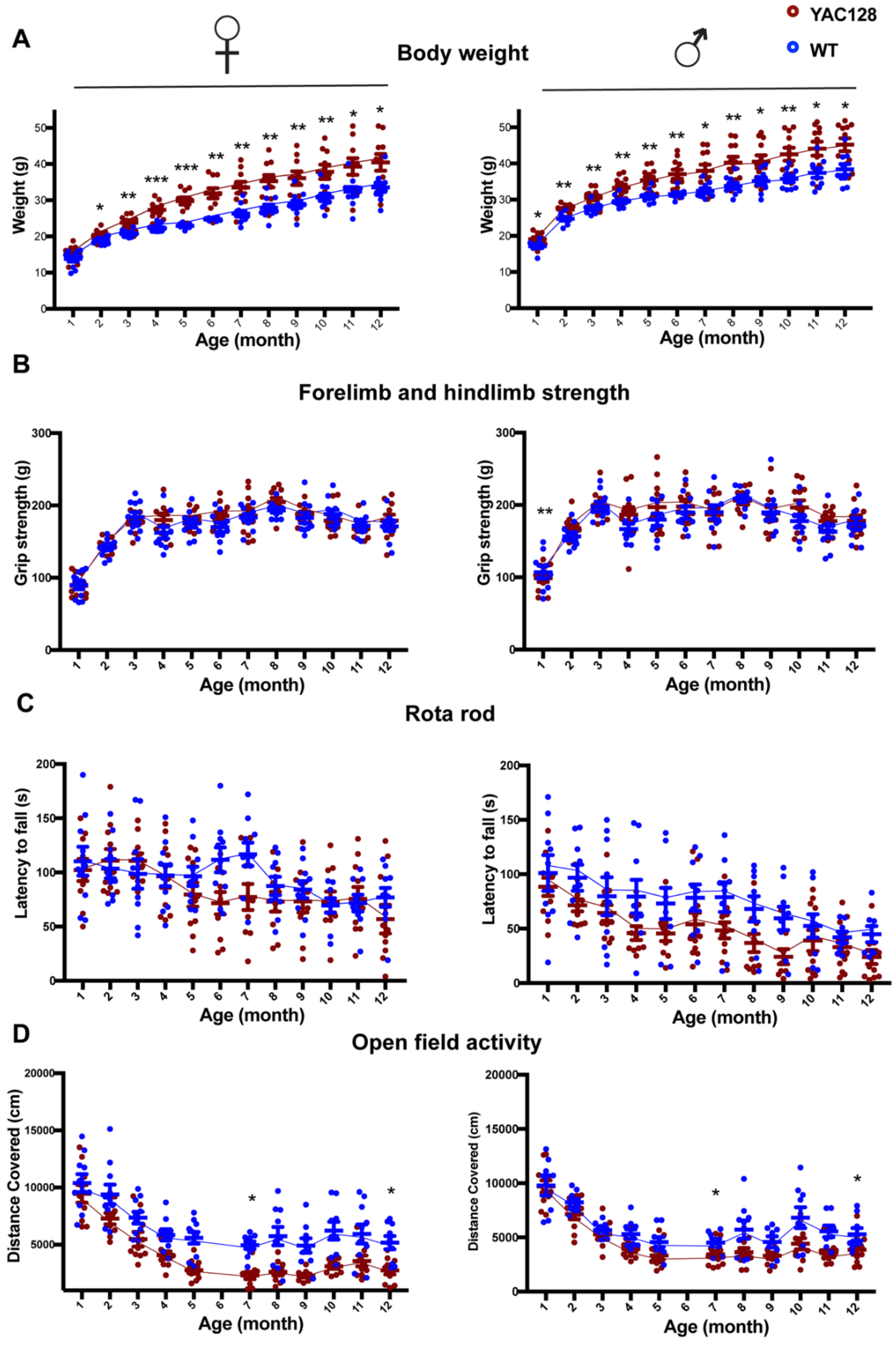

Supplementary Figure 9. YAC128 mice gained weight faster than their wild-type littermates, but did not develop motor phenotypes up to 12 month of age.

(**A**) Both YAC128 males and females gained weight faster than their wild-type counterparts: males [F(genotype)$_{1,236}$ = 4.473, *p* = 0.036], females [F(genotype)$_{1,211}$ = 120.34, *p*= 0.001]. In both cases, YAC128 and wild-type mice gained weight with age: males [F(age)$_{11,236}$ = 50.129, *p* < 0.001], females [F(age)$_{11,211}$ = 61.347, *p* < 0.001], but there was no difference between genotypes in the change in weight gain over the 12-month period: males [F(genotype x age)$_{11,236}$ = 0.994, *p* = 0.453], females [F(genotype x age)$_{11,211}$ = 1.787, *p* = 0.058]. **(B)** Grip strength (fore- and hind-limbs) increased with age for both YAC128 and wild-type mice: males [F(age)$_{11,235}$ = 18.765, *p* < 0.001], females [F(age)$_{11,234}$ = 24.547, *p* < 0.001]. Whilst the grip strength between wild-type and YAC128 males did differ [F(genotype)$_{1,235}$ = 4.124, *p* = 0.044], this was only at 1 month of age and there was no difference in the grip strength between YAC128 and wild-type females [F(genotype)$_{1,234}$ = 0.633, *p* = 0.427]. There was no difference in the change in grip strength between YAC128 and wild-type mice up to 12 months: males [F(genotype x age)$_{11,235}$ = 0.785, *p* = 0.655], females [F(genotype x age)$_{11,234}$ = 0.828, *p* = 0.612]. **(C)** Rotarod performance changed little with age for both YAC128 and wild-type mice: males [F(age)$_{11,230}$ = 0.683, *p* = 0.754], females [F(age)$_{11,232}$ = 0.662, *p* = 0.773]. There was a difference in rotarod performance between YAC128 and wild-type males F(genotype)$_{1,230}$ = 9.928, *p* = 0.002], but *post-hoc* analysis with weight as a co-variate indicated that a genotype-specific difference had not occurred. There was no difference in the rotarod performance between YAC128 and wild-type females [F(genotype)$_{1,232}$ = 0.327, *p* = 0.568] and no difference in the change in rotarod performance between YAC128 and wild-type mice over the 12 month period: males [F(genotype x age)$_{11,230}$ = 0.356, *p* = 0.948], females [F(genotype x age)$_{11,232}$ = 1.019, *p* = 0.431]. **(D)** The total distance travelled during exploratory activity decreased for both YAC128 and wild-type mice over the course of the 12 months: males [F(age)$_{10,204}$ = 11.455, *p* < 0.001], females [F(age)$_{10,206}$ = 9.507, *p* < 0.001]. Whilst genotype differences were apparent: males [F(genotype)$_{1,204}$ = 23.365, *p* < 0.001], females [F(genotype)$_{1,206}$ = 47.651, *p* < 0.001], these were not progressive: males [F(genotype x age)$_{10,204}$ = 0.529, *p* = 0.868], females [F(genotype x age)$_{10,206}$ = 0.506, *p* = 0.885]. *Post-hoc* analysis with weight as a covariate found no consistent differences in the total distance travelled between wild-type and YAC128 mice. YAC128 male (n = 11), wild-type male (n = 9), YAC128 female (n = 10), wild-type female (n = 10). Statistical analysis was GLM ANOVA or ANCOVA with Bonferroni *post hoc* correction *p ≤ 0.05, **p ≤ 0.01, ***p ≤ 0.001.

Supplementary Figure 10

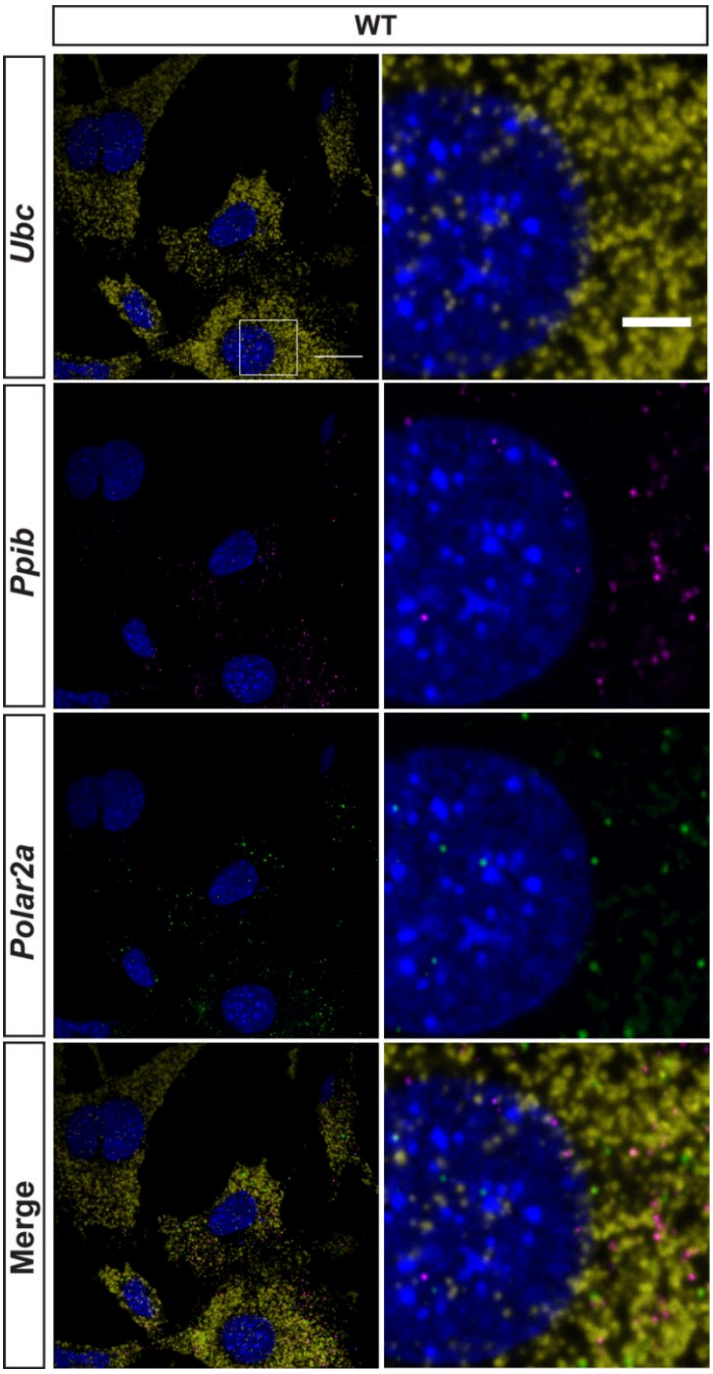

Supplementary Figure 10. Detection of mRNA transcripts of three differentially expressed housekeeping genes in wild-type MEFs.

Wild-type MEFs were stained with a set of probes recognising housekeeping mRNAs. *Ubc* is a highly expressed transcript, *Ppib* is expressed at high to medium levels whereas *Polar2a* is an mRNA of low abundantance. Note that most of the transcripts are localised outside of the nucleus. Nuclei were stained with DAPI (blue). Wild type (n = 3). N corresponds to biological replicates; MEF cell lines were derived from different animals. Scale bar = 20 µm in the main image (left-hand side) and 5 µm in the cropped magnified image (right-hand side). WT = wild type.

Supplementary Figure 11

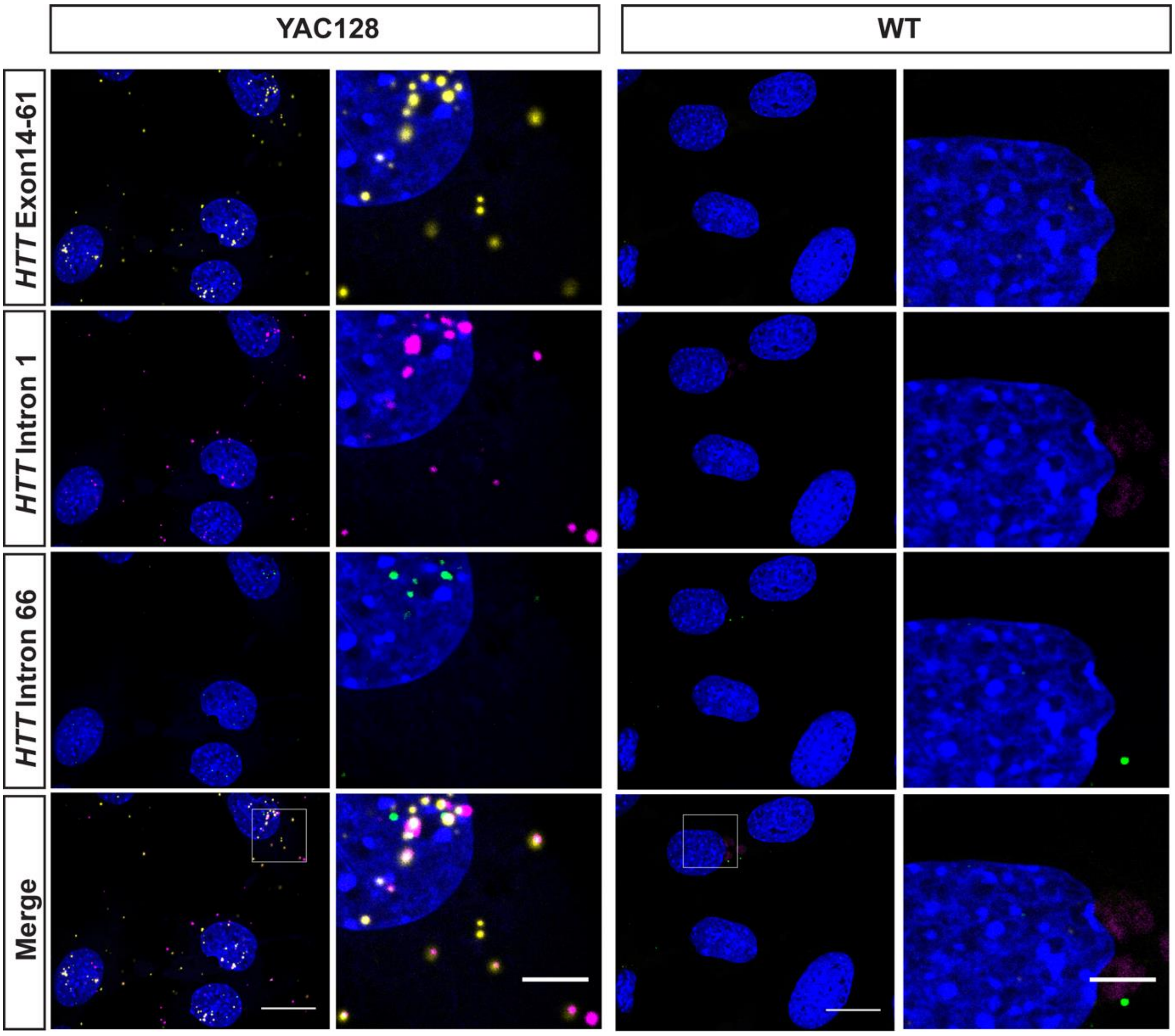

Supplementary Figure 11. Subcellular distribution of human *FL-HTT* and *HTT1a* transcripts in the YAC128 MEFs.

Full-length *HTT* (yellow) and *HTT1a* (magenta) transcripts were located in both the nucleus and cytoplasm in YAC128 MEFs. They were often co-localised in small nuclear RNA clusters and were present in the cytoplasm as single transcripts. The *HTTintron66* (green) probe visualised pre-mRNA which was only detected in the nucleus, where it occasionally co-localised with the *HTT* and *HTT1a* transcripts. Wild-type sections showed no staining with any of the human huntingtin probes. Nuclei were stained with DAPI (blue). YAC128 (n = 3), wild type (n = 3). N corresponds to biological replicates; MEF cell lines were derived from different animals. Scale bar = 20 µm in the main image (left-hand side) and 5 µm in the cropped magnified image (right-hand panel). WT = wild type.

Materials and Methods

Cell culture and ASO transfection

Mouse embryonic fibroblasts (MEFs) were isolated from E14.5 YAC128 embryos and maintained in DMEM supplemented with 10% foetal bovine serum (FBS) and 1% penicillin-streptomycin in a humidified incubator at 37˚C with 5 % CO_2. They were passaged approximately once a week when cells reached around 95 % confluence. MEFs were transformed with the viral oncogene Simian Virus-40 large tumour antigen (SV40 T-antigen) using the Cell Immortalization Kit (CILV01 Alstem). Briefly, 2 x 10^5 cells were seeded in a 6-well plate and infected with 10 µl SV40 and 1 µl trans plus viral enhancer (V050) in 1 mL of DMEM (with 10% FBS and 1% penicillin-streptomycin) overnight. Next day, the medium containing the virus was replaced and the cells were allowed to recover for 72 h. Cells that had integrated the virus were selected with puromycin (Thermo Fisher Scientific) at 5 µg/mL for the initial screen and then at 7 µg/mL for later passages. To isolate colonies from single cells, the cell suspension was serially diluted and cultured in 96 wells plates. MEFs were screened for SV40 T-antigen integration by PCR as recommended by the manufacturer (Alstem) and western blot.[1] Three YAC128 and three wild-type cell clones were chosen for downstream applications.

MEFs were seeded in 6- or 12-well plates (for qPCR experiments), in 24-well plates (for RNAscope) or in 96-well plates (for QuantiGene analysis) at a density of 1.5 x 10^6 cells/cm^2 in DMEM/6% FBS (QuantiGene) or at a density of 5 x 10^5 cells/cm^2 in DMEM/10% FBS (RNAscope). Treatment with ASOs was conducted one day after plating, when the medium was substituted with DMEM/OptiMEM (in a 1:1 ratio)/ 3% FBS before ASO transfection. Cells were transfected with 2, 20 or 200 nM ASO (437527), targeting mouse and human huntingtin, or 2, 20 or 200 nM non-targeting negative control ASO (676630) using 2.5

nL of DharamFECT 1 (Horizon Discovery) per µL of total media per well, as per the manufacturer’s recommendations.

Two days after transfection, MEFs were used either for QuaniGene or qPCR analysis. For qPCR experiments, the cells were washed in PBS after media aspiration and lysed in Qiazol lysis reagent (Qiagen). The cells were scraped from the bottom of the wells, transferred to a new tube and snap-frozen on dry ice.

Relative real-time quantitative PCR (qPCR)

Supplementary Table 1. Design of qPCR assays for the detection of human *HTT* transcripts

**Name**	**Sequence 5'→ 3'**	**Primer Efficiency**	**Probe dye/quencher**
3'UTR *HTT* Fwd	GCTGTCCTGCAGTAGAAGGT	1.96	
3'UTR *HTT* Rev	CATGGCATCTGTGCATCCAG		
3'UTR *HTT* Probe	CTTTGGGAACACTGGCCTGGGTCTCCCTG		Texas Red/BHQ2
Intron 56 *HTT* Fwd	CTATTCACAGAGGTGGGAGC	1.93	
Intron 56 *HTT* Rev	GGTCTTGGGGATTTGTCACT		
Intron 56 *HTT* Probe	TGACAGAGTCCAGCTTGCCCACTGGC		Texas Red/BHQ2
PolyA2_$_{1}$ *HTT* Fwd	AGCACAGATGAAAAACAAAGCCCT	1.90	
PolyA2_$_{1}$ *HTT* Rev	GGGAAGAAGACACAAGAAACACTT		
PolyA2_$_{1}$ *HTT* Probe	TTGCAAGTCTGTCATCTTTGTCTAACTTCCTA		Texas Red/BHQ2

Tissue lysis and QuantiGene assays

Supplementary Table 2 Design of QuantiGene assays for the detection of human *HTT* transcripts.

Transcript/Gene symbol	Transcript/Gene name	Accession number	Probe set region
HTT PolyA1	Huntingtin intron 1 polyA1	GS03195	227-2625
HTT PolyA1A2	Huntingtin intron 1 polyA1A2	GS03129	580-4566
HTT Intron 1_3’	Huntingtin intron 1_3’	GS03130	1024-1556
HTT Intron 3	Huntingtin intron 3	GS03131	1251-1692
HTT Intron 56	Huntingtin intron 56	GS03277	57-389
HTT-FL (exon 43-46)	Full-length human huntingtin mRNA	NM_002111	5891-6331
HTT-FL (3’UTR)	Ful- length human huntingtin mRNA	NM_002111	9720-10131
Htt-FL (exon 50-53)	Full-length mouse huntingtin mRNA	NM_010414	6901-7433
Htt-FL (short_3’UTR)	Full-length mouse huntingtin mRNA	NM_010414	9553-9993
Htt-FL (long_3’UTR)	Full-length mouse huntingtin mRNA	NM_010414	12640-13104
Canx	Calnexin	NM_007597	76-727
Rpl13a	Ribosomal protein L13a	NM_009438	2-467
Ubc	Ubiquitin C	NM_019629	113-676
Atp5b	ATP synthase subunit beta	NM_016774	22-409
Eif4a2	Eukaryotic translation initiation factor 4A2	NM_013506	710-1271

RNAscope analysis and quantification

Brains hemispheres from 2-month-old YAC128 mice were harvested, embedded in optimal cutting temperature compound (OCT, CellPath Ltd) and stored at -80 °C until further use. Brain blocks were equilibrated to about -30°C in a cryostat (Bright Instruments), cut into 20 µm thick sagittal sections and mounted onto SuperfrostPlus slides (VWR, 25 x 75 x 1.0 mm). The brain sections, or cells cultured on coverslips, were fixed in 4% paraformaldehyde (Pioneer Research Chemical Ltd) for 20 min at 4°C followed by dehydration in an increasing concentration of ethanol, starting at 50%, moving to 70% and finishing with 100%, each time for 5 min (for brain sections) or 1 min (for cultured cells) at room temperature. Next, a hydrophobic barrier

was created around each section with a hydrophobic barrier pen (ImmEdge Pen, Vector; H-4000) and allowed to air-dry for about 1 min. About five drops of 3% hydrogen peroxide solution (H_2O_2) was added onto the section and incubated for 10 min at room temperature, followed by three washes with PBS. Protease IV (for brain sections) or protease III (for cultured cells, diluted 1:15) was then added to the samples and incubated for 30 min (for brain sections) or 10 min (for cultured cells) at room temperature. The protease was removed, and the slides submerged in PBS three times for 5 min each.

The hybridization and amplification steps were performed in accordance with the RNAscope protocol (Advanced Cell Diagnostics, Inc.). RNAscope probes were: full-length human *HTT* exons 14-61 (473201), and exons 7–13 (842001), intron 1 human *HTT* (561431), intron 66 human *HTT* (493761), full-length mouse *Htt* (424681), intron 1 mouse *Htt* (419071) and intron 2 mouse *Htt* (445571). Probes were hybridized for 2 h at 40°C in a HybEZ oven (Advanced Cell Diagnostics, Inc., 220 VAC, Cat. No. 310013). Following each amplification step, the slides were washed twice with wash buffer for 5 min each at room temperature. Each probe was developed in three steps: the HRP (horse radish peroxidase) step, the TSA (tyramide signal amplification) fluorophore step and the HRP blocker step, using the RNAscope Multiplex Fluorescent v2 Assay. Each step was followed by two washes in wash buffer for 5 min at room temperature. A different TSA fluorophore (PerkinElmer) was assigned to each probe channel. The following fluorophore dilutions were used: fluorescein 1:1500, Cy3 1:1500, Cy5 1:500 or 1:750. Subsequently, the slides were counterstained with DAPI for about 5 min at room temperature and mounted in VECTASHIELD Vibrance Antifade Mounting Media (Vector Laboratories). A 24 x 50 mm glass coverslip (VWR International) was placed over the tissue section and the slides were air dried before being imaged. Images were acquired with an upright (Leica SP8vis) or inverted (Zeiss LSM980) confocal integrated microscope system with a 63x objective (HC PL APO 63x/1.40 Oil).

To analyse the intracellular localisation of huntingtin transcripts a custom macro in FIJI (ImageJ, Image Analysis) was written. Images were converted to 8 bit before positive signals for DAPI and for each transcript were identified using a threshold analysis of their signal intensity: DAPI 12/255, *FL-HTT* 25/255, *HTT1a* 12/255 or *FL-Htt* 35/255. Objects that were identified but contained less than 5 pixels were ignored. The DAPIsignal was used to create a mask for cell nuclei, to separate out nuclear and cytoplasmic signals for the huntingtin transcripts. Eighteen images containing on average three to four cells were analysed. The total area of pixels located inside and outside of the masked nucleus was calculated for each transcript (*FL-HTT*, *HTT1a* or *FL-Htt*) in each image. The results were depicted as a % ratio of nuclear and cytoplasmic huntingtin transcripts.

Colocalisation analysis

Colocalisation analysis of human full-length *HTT* and *HTT*1a mRNAs or full-length human and mouse huntingtin transcripts was performed using Fiji software. First, the background was measured and subtracted for all images. Then, images were analysed using the Colocalisation Threshold plugin. The Pearson correlation coefficient (Rcoloc), which measures pixels that are colocalised above both the channel thresholds, was retrieved. Subsequently, the Colocalisation Test, which computes Rrand value, was performed. Rrand determines the extent of colocalization between two images when pixel values of the second image are randomly reshuffled. Rcoloc and Rrand were plotted in GraphPad 9 software.

Antibodies

Supplementary Table 3. Source of antibodies used for HTRF, immunohistochemistry and western blotting.

Name	Immunogen	Epitope	Species	Reference / Source
2B7	HTT peptide: aa 1-17		Mouse Monoclonal	CHDI Foundation[2]
MW1	HTT Exon1 (67Q)	PolyQ	Mouse Monoclonal	CHDI Foundation[3]
4C9	HTT peptide: aa 51-71		Mouse Monoclonal	CHDI Foundation[4]
MW8	HTT Exon1 (67Q)	aa 83-90	Mouse Monoclonal	CHDI Foundation[3]
MAB5490	Recombinant HTT: aa 115-129		Mouse Monoclonal	Sigma-Aldrich, MAB5490
MAB2166	HTT fusion protein: aa 181-810	aa 443-457	Mouse Monoclonal	Sigma-Aldrich, MAB2166
D7F7	HTT peptide:	residues surrounding Pro1220	Rabbit Monoclonal	Cell Signaling Technology #5656
S830	Exon 1 HTT with 53Q		Sheep Polyclonal	In-house[5]
3B5H10	PolyQ	PolyQ	Mouse Monoclonal	Sigma-Aldrich P1874
CHDI-90000148			Rabbit Polyclonal	CHDI Foundation This paper
SV40 T-antigen			Mouse monoclonal	Abcam Ab16879

Homogeneous Time-Resolved FRET (HTRF)

HTRF was performed as previously described.[6] Briefly, individual brain regions were homogenised (w/v) in ice-cold bioassay buffer [PBS, 1% Triton-X-100] supplemented with complete protease inhibitor cocktail tablets (Roche), using a Fast-Prep-24 instrument (MP Biomedicals). Crude lysates were used for HTT aggregation assays, whereas for soluble HTT assays the supernatant was used following centrifugation (3500x*g* for 10 min). For HTRF assay optimisations, antibody concentrations were established by maintaining the terbium donor at 1 ng/well, whilst titrating the d2 acceptor at either 1, 2, 5, 10, 20 or 40 ng/well (Supplementary Fig. 12A). At the same time a 4-point lysate titration was performed (Supplementary Fig. 12B) by diluting YAC128 cortical homogenates with either age-matched wild-type homogenates at 2 months of age for soluble mutant HTT assays or 12-months of age for HTT aggregation assays. Dilutions were performed with lysis buffer for the total soluble full-length HTT assay only. Antibody and lysate concentrations were chosen that resulted in a titration curve that was as close to linear as possible (Supplementary Table 4).

For YAC128 and wild-type MEFs, HTRF assays were optimised exactly as for tissues except that a defined cell number was lysed from a frozen cell pellet in ice-cold bioassay buffer [PBS, 1% Triton-X-100] supplemented with complete protease inhibitor cocktail tablets (Roche), by sequential pipetting and a vortex followed by a freeze-thaw step on dry ice Supplementary Fig. 13. The selected antibody concentration are summarised in Supplementary Table 5.

HTRF assays were performed in triplicate: 5 µL of optimised antibody concentration in HTRF detection buffer [50 mM Nah_2PO_4, 0.2 M KF, 0.1% bovine serum albumin, 0.05% Tween-20] was added to 10 µL of the optimised lysate concentration into a 384-well proxiplate (Greiner Bio-One). Following incubation for 2 h on an orbital shaker (250 rpm), plates were read using an EnVision (Perkin Elmer) plate reader.

Supplementary Figure 12

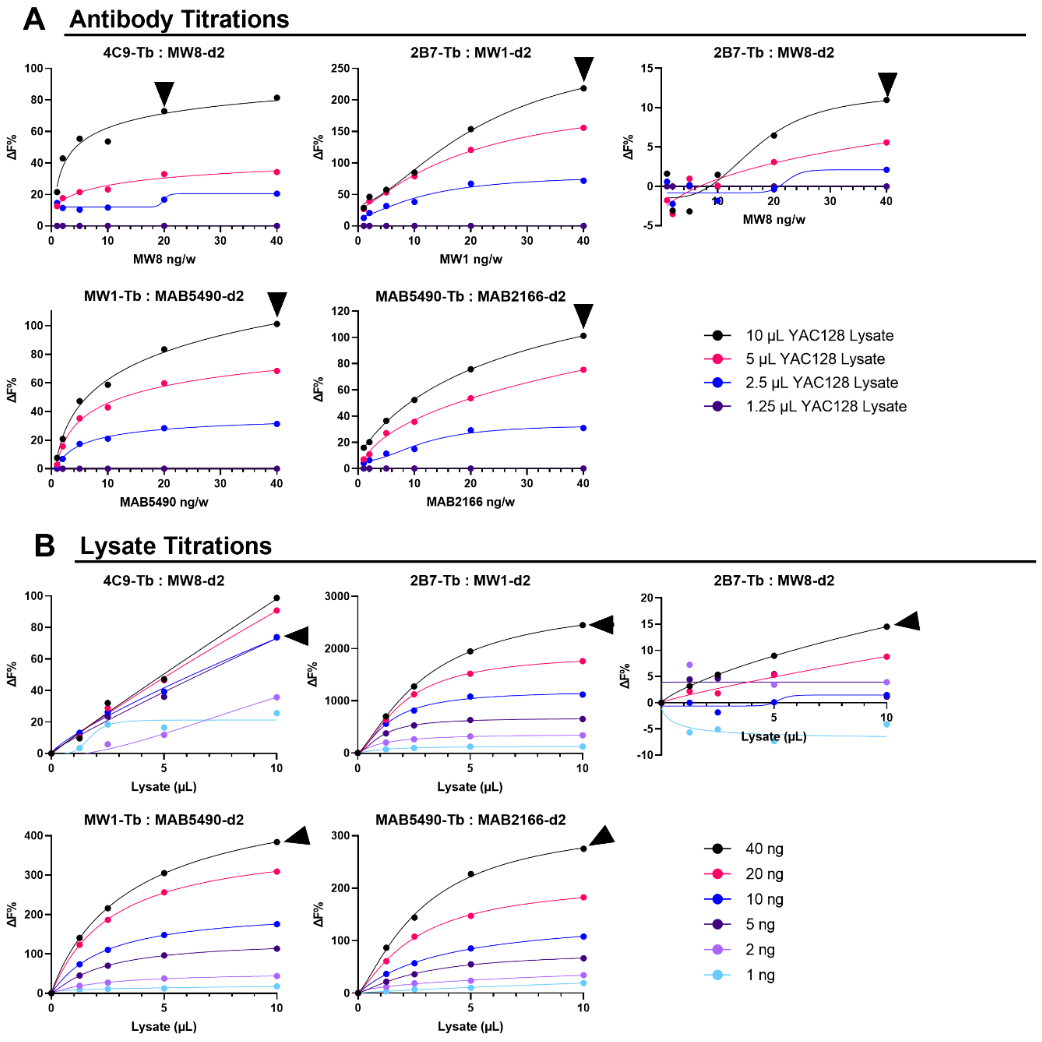

Supplementary Figure 12. Optimisation of HTRF assays for use with YAC128 tissue lysates.

Optimisation of HTRF assays was performed on cortical lysates from either 12-month-old YAC128 mice for the 4C9-MW8 assay or on 2-month-old cortical lysates for the soluble HTT assays as, at the age range under investigation, these have the greatest concentration of aggregated HTT and soluble HTT, respectively. To optimise the antibody and lysate concentrations, a checker-board matrix was performed. In one dimension, the donor antibody was kept constant at 1 ng / well, and the acceptor antibody was titrated from 1 ng / well to 40 ng / well. In the second dimension, two-fold serial dilutions of cortical lysates from YAC128 mice of 1.25 – 10 µL were performed by diluting with age-matched wild type lysate for all assays except MAB5490-MAB2166, for which the dilutions were performed with lysis buffer. **(A)** The maximum concentration prior to saturation was chosen as optimal (arrow). **(B)** The change in fluorescent signal with lysate dilution for a given antibody concentration. The curve corresponding to the optimal antibody concentration is indicated (arrow). The lysate concentration chosen was that which would allow for an increase in aggregated HTT or decrease in soluble HTT to fall within the linear range of the assay, as far as was possible. The change in fluorescent signal is denoted as ΔF%. Ng/w = ng / well.

Supplementary Figure 13.

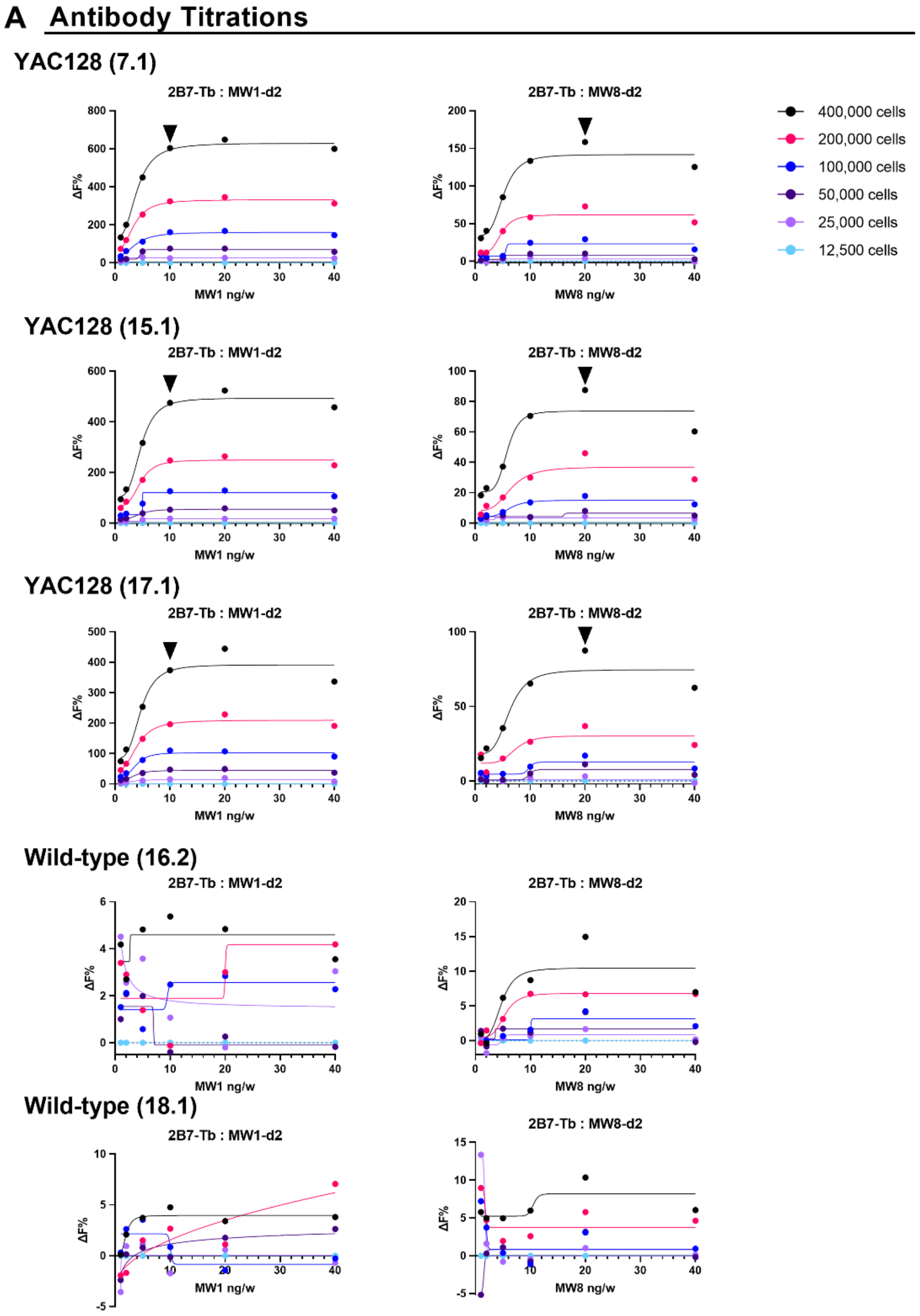

Supplementary Figure 13. Optimisation of HTRF assays for use with YAC128 MEFS. Optimisation of HTRF assays was performed on YAC128 (lines 7.1, 15.1 and 17.1) and wild-type (lines 16.2 and 18.1) MEFs. To optimise the antibody and lysate concentrations, a checker-board matrix was performed. In one dimension, the donor antibody was kept constant at 1 ng / well, and the acceptor antibody was titrated from 1 ng / well to 40 ng / well. In the second dimension, two-fold serial dilutions from 400,000 to 12,500 cells was performed by diluting with lysis buffer. **(A)** The maximum concentration prior to saturation was chosen as optimal (arrow). The change in fluorescent signal is denoted as ΔF%. Ng/w = ng / well.

Supplementary Figure 13.

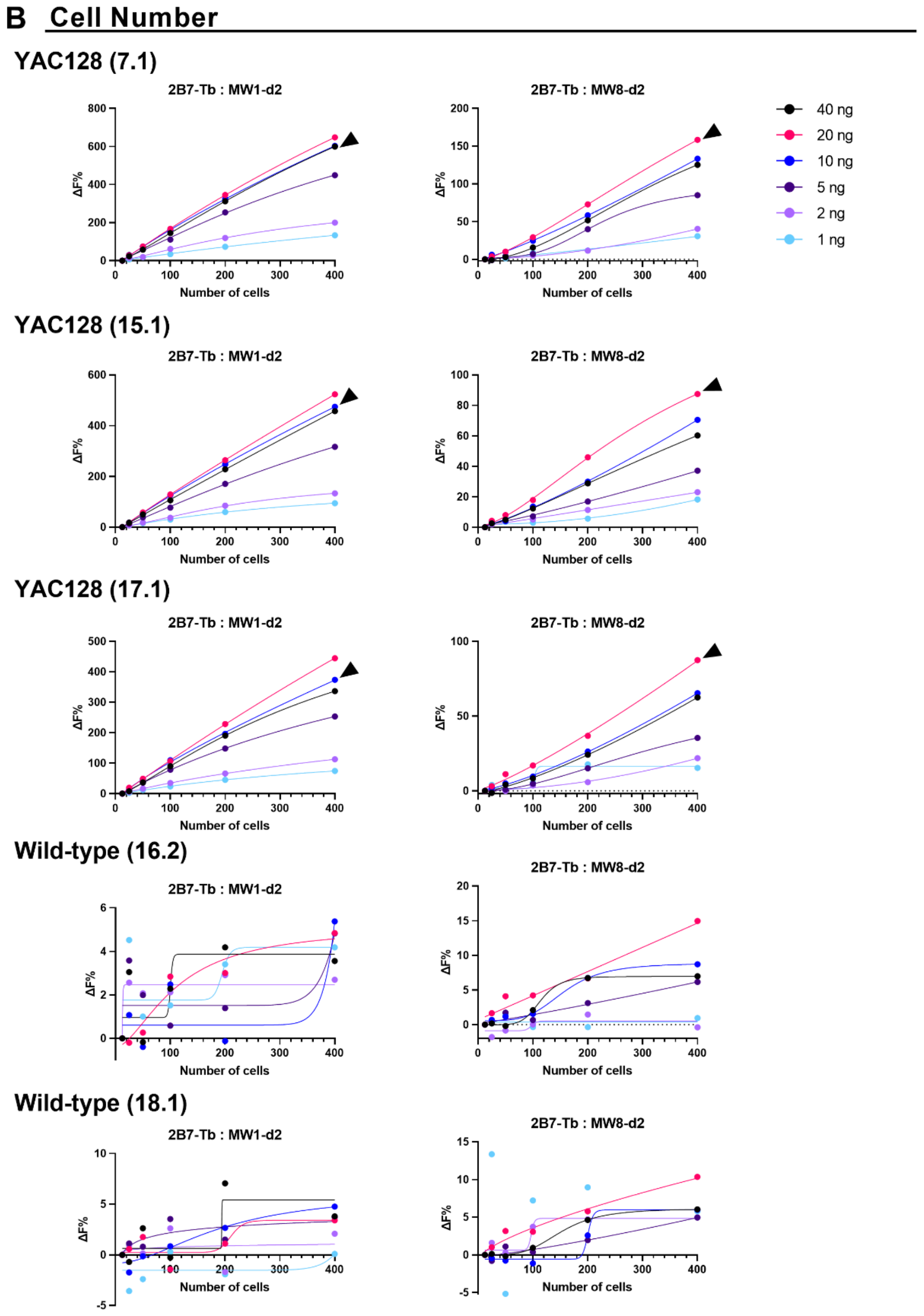

Supplementary Figure 13. Optimisation of HTRF assays for use with YAC128 MEFS. (B) The change in fluorescent signal with cell number for a given antibody concentration. All assays were run with 400,000 cells for this investigation. The change in fluorescent signal is denoted as ΔF%. Ng/w = ng / well.

Supplementary Table 4 HTRF assay conditions selected for use with YAC128 tissue lysates.

Antibody pairing (Tb donor : d2 acceptor)	Antibody (ng/well)	Amount of Lysate (µL/well) (fold-dilution)
4C9–MW8	20 ng	10 µL
2B7–MW8	40 ng	10 µL
MW1–MAB5490	40 ng	5 µL (1 : 2)
MAB5490–MAB2166	40 ng	5 µL (1 : 2)

Supplementary Table 5 HTRF assay conditions selected for use with YAC128 MEFs.

Antibody pairing (Tb donor : d2 acceptor)	Antibody (ng/well)	Number of cell used / well
2B7-MW1	10 ng	400,000
2B7–MW8	20 ng	400,000
D7F7-MAB5490	40 ng	400,000

References

[1] Landles C, Milton RE, Ali N, Flomen R, Flower M, Schindler F, Gomez-Paredes C, Bondulich MK, Osborne GF, Goodwin D, Salsbury G, Benn CL, Sathasivam K, Smith EJ, Tabrizi SJ, Wanker EE, Bates GP: Subcellular Localization And Formation Of Huntingtin Aggregates Correlates With Symptom Onset And Progression In A Huntington's Disease Model. Brain Commun 2020, 2:fcaa066.

[2] Weiss A, Abramowski D, Bibel M, Bodner R, Chopra V, DiFiglia M, Fox J, Kegel K, Klein C, Grueninger S, Hersch S, Housman D, Regulier E, Rosas HD, Stefani M, Zeitlin S, Bilbe G, Paganetti P: Single-step detection of mutant huntingtin in animal and human tissues: A bioassay for Huntington's disease. Analyt Biochem 2009, 395:8-15.

[3] Ko J, Ou S, Patterson PH: New anti-huntingtin monoclonal antibodies: implications for huntingtin conformation and its binding proteins. Brain Res Bull 2001, 56:319-29.

[4] Landles C, Sathasivam K, Weiss A, Woodman B, Moffitt H, Finkbeiner S, Sun B, Gafni J, Ellerby LM, Trottier Y, Richards WG, Osmand A, Paganetti P, Bates GP: Proteolysis of mutant huntingtin produces an exon 1 fragment that accumulates as an aggregated protein in neuronal nuclei in Huntington disease. J Biol Chem 2010, 285:8808-23.

[5] Sathasivam K, Woodman B, Mahal A, Bertaux F, Wanker EE, Shima DT, Bates GP: Centrosome disorganization in fibroblast cultures derived from R6/2 Huntington's disease (HD) transgenic mice and HD patients. Hum Mol Genet 2001, 10:2425-35.

[6] Landles C, Milton RE, Jean A, McLarnon S, McAteer SJ, Taxy BA, Osborne GF, Zhang C, Duan W, Howland D, Bates GP: Development of novel bioassays to detect soluble and aggregated Huntingtin proteins on three technology platforms. Brain Commun 2021, 3:fcaa231.
